# MCL1 and BCL-xL Levels in Solid Tumors Are Predictive of Dinaciclib-Induced Apoptosis

**DOI:** 10.1371/journal.pone.0108371

**Published:** 2014-10-07

**Authors:** Robert N. Booher, Harold Hatch, Brian M. Dolinski, Thi Nguyen, Lauren Harmonay, Ali-Samer Al-Assaad, Mark Ayers, Michael Nebozhyn, Andrey Loboda, Heather A. Hirsch, Theresa Zhang, Bin Shi, Carrie E. Merkel, Minilik H. Angagaw, Yaolin Wang, Brian J. Long, Xianlu Q. Lennon, Nathan Miselis, Vincenzo Pucci, James W. Monahan, Junghoon Lee, Anna Georgieva Kondic, Eun Kyung Im, David Mauro, Rebecca Blanchard, Gary Gilliland, Stephen E. Fawell, Leigh Zawel, Alwin G. Schuller, Peter Strack

**Affiliations:** 1 Discovery Oncology, Merck Research Laboratories, Boston, Massachusetts, United States of America; 2 BioInformatics, Merck Research Laboratories, Boston, Massachusetts, United States of America; 3 In vivo Pharmacology, Merck Research Laboratories, Boston, Massachusetts, United States of America; 4 Biomarkers, Merck Research Laboratories, Boston, Massachusetts, United States of America; 5 Pharmacokinetics, Pharmacodynamics and Drug Metabolism, Merck Research Laboratories, Boston, Massachusetts, United States of America; 6 Quantitative Pharmacology and Pharmacometrics, Merck Research Laboratories, Boston, Massachusetts, United States of America; 7 Clinical Oncology, Merck Research Laboratories, Boston, Massachusetts, United States of America; Roswell Park Cancer Institute, United States of America

## Abstract

Dinaciclib is a potent CDK1, 2, 5 and 9 inhibitor being developed for the treatment of cancer. Additional understanding of antitumor mechanisms and identification of predictive biomarkers are important for its clinical development. Here we demonstrate that while dinaciclib can effectively block cell cycle progression, *in vitro* and *in vivo* studies, coupled with mouse and human pharmacokinetics, support a model whereby induction of apoptosis is a main mechanism of dinaciclib's antitumor effect and relevant to the clinical duration of exposure. This was further underscored by kinetics of dinaciclib-induced downregulation of the antiapoptotic *BCL2* family member *MCL1* and correlation of sensitivity with the *MCL1*-to-*BCL-xL* mRNA ratio or *MCL1* amplification in solid tumor models *in vitro* and *in vivo*. This MCL1-dependent apoptotic mechanism was additionally supported by synergy with the BCL2, BCL-xL and BCL-w inhibitor navitoclax (ABT-263). These results provide the rationale for investigating *MCL1* and *BCL-xL* as predictive biomarkers for dinaciclib antitumor response and testing combinations with BCL2 family member inhibitors.

## Introduction

Recent clinical activity of cyclin-dependent kinase (CDK) inhibitors has re-invigorated interest in their application for the treatment of cancer [1], [2]. The CDK inhibitor dinaciclib, in particular, exhibited a positive response rate in chronic lymphocytic leukemia (CLL) Phase 1 trials, however, responses have been less pronounced in other non-biomarker selected single-agent solid-tumor clinical trials [3]–[6]. As such, a deeper understanding of this CDK inhibitor's mechanism of action and methods for identifying solid tumors most likely to respond will benefit its clinical development. Dinaciclib potently inhibits CDKs 1, 2, 5 and 9, which are involved in a variety of cellular processes including cell cycle regulation and RNA polymerase II-based (RNAPII) transcription [2], [7]. In agreement with this, *in vitro* studies have shown its ability to induce cell cycle arrest and apoptosis resulting in potent cell-killing across a variety of cancer types using a 7 day clonogenicity assay [7]. Dinaciclib blocks the cell cycle through inhibition of CDKs 1 and 2 and represses transcription through inhibition of CDK9, which phosphorylates the carboxyl-terminal repeat domains (CTD) of RNAPII [8], [9]. Transcriptional repression results in the rapid downregulation of mRNA transcripts and proteins with short half-lives, such as the antiapoptotic BCL2 family member MCL1, and induces differential levels of apoptosis [10], [11]. Other pan-CDK inhibitors, such as flavopiridol, CYC202 (R-roscovitin) and SNS-032, have also been reported to block transcription and downregulate MCL1 [12]–[14]. These studies have hypothesized that apoptosis rather than cell cycle repression may be the major mechanism-of-action for such CDK inhibitors based on the rapid apoptotic induction response of CLL samples treated *ex vivo* and high clinical response rate in CLL. As such, while antiapoptotic proteins may be one means of influencing dinaciclib-directed cell-killing, other mechanisms of dinaciclib sensitivity and resistance have been described including the unfolded protein response and pathway status of Notch, Transforming Growth Factor-beta (TGF-β) and p53 [15]–[17].

In exploring the mechanism of dinaciclib's therapeutic effect, clinically relevant concentrations and duration of exposure need to be considered. In patients, dinaciclib is administered by 2 hour (hr) intravenous (i.v.) infusion, and reaches maximum concentrations (C_max_) approximately 2 hr after initiation of the infusion with rapid elimination after the infusion is stopped [5], [6], [18]. At recommended Phase 2 doses (RP2D), dinaciclib plasma concentrations are maintained above concentrations shown to be effective preclinically (≥50 nM) for 6–8 hr [5]–[7], [18]. We conducted *in vitro* studies using dinaciclib treatments spanning this concentration range for durations of 2–24 hr as compared to longer term (3–7 days) historical durations associated with non-discriminate cancer cell-killing. We also evaluated biomarkers of sensitivity and mechanisms of cell-killing identified from *in vitro* studies in seven xenograft models. Here we demonstrate using both *in vitro* and *in vivo* models that, at clinically relevant concentrations and durations of exposure, induction of tumor cell apoptosis is a major mechanism of dinaciclib's effect and not cell cycle inhibition. While apoptosis alone may not be the sole mechanism of dinaciclib's *in vivo* effects, the data presented here support *MCL1* amplification or the *MCL1:BCL-xL* mRNA ratio as a means of enriching for patients more likely to respond to this treatment.

## Material and Methods

### Chemicals and cell culture

Dinaciclib (MK-7965, formerly SCH 727965, Merck, Whitehouse Station, NJ); paclitaxel (LC Laboratories, Woburn, MA); triptolide (Sigma); navitoclax (ABT-263) (Selleck Chemicals); KDR inhibitor B [19] (Merck, Whitehouse Station, NJ). For *in vitro* studies, compounds were dissolved in dimethylsulfoxide (DMSO, Sigma-Aldrich) at 10 mmol/L and aliquots were stored at −80°C. The final concentration of DMSO in all cellular experiments was ≤0.2%. Cell lines were obtained from American Type Culture Collection (ATCC, Manassas, VA), Leibniz Institute DSMZ-German Collection of Microorganisms and Cell Cultures (DSMZ, Braunschweig, Germany), Japanese Collection of Research Bioresources Cell Bank (JCRB, Japan), European Collection of Cell Cultures (ECACC), Sigma-Aldrich (St. Louis, MO) or as described in Table S5.

### Immunoblot and immunohistochemistry

Whole cell lysates from *in vitro* cultured cells were prepared by washing the cells with cold PBS and lysing in cold RIPA buffer (Cell Signaling Technology) according to the manufacturer. The RIPA buffer contained one protease (complete, mini, EDTA-free, Roche) and one phosphatase (PhosSTOP, Roche) inhibitor cocktail tablet per 10 ml. In treatments that caused adherent cell detachment, the detached cells were collected by centrifugation, washed with PBS and pooled with the lysate prepared from the attached cells. Whole cell lysates from xenograft tumor pieces (previously snap-frozen and stored at −80°C) were prepared by disruption/homogenation at 4°C in a 2 ml conical-end microfuge tube containing two stainless steel beads (5/32″, grade 25, Ball Supply Corp) and 0.4 ml lysis buffer (1% Triton X-100, 30 mM Tris pH 7.4, 1 mM EDTA and protease and phosphatase cocktail inhibitors) using a Qiagen TissueLyser II, shaking at 30 Hz for 2 min. 200 µl of pre-chilled 3X RIPA containing protease and phosphatase cocktail inhibitors was added to the homogenate, rotated at 4°C for 10 min. Lysates from in vitro cultured cells and tumor pieces were clarified by microcentrifugation at 12,000 rpm for 10 min at 4°C. The supernatant was removed, normalized for protein content using a BCA kit (Pierce, Rockford, IL) and stored at −80°C. In general, 5 µg of lysate were subjected to SDS-PAGE except for cleaved PARP analysis, which in certain cases, required loading of 0.5 µg of lysate. The mouse anti-RNAPII antibodies (8WG16, H5 and H14) were from Covance (Emeryville, CA); rabbit polyclonal antibodies against MCL1 (#5453), full length PARP (F.L. PARP #9546) and cleaved PARP (#9541); rabbit monoclonal antibodies (mAbs) against BCL2 (#2870), BCL-xL (#2764), and mouse mAbs against α-tubulin (#3873) were from Cell Signaling (Beverly, MA). Secondary HRP-labeled goat anti-rabbit (#7074) and horse anti-mouse antibodies (#7076) (Cell Signaling, Beverly, MA) in conjunction with SuperSignal West Pico or Femto Chemiluminescent Substrate (Thermo Scientific) were used for protein detection on autoradiography film (BioMax MR, Carestream Kodak, Rochester, NY). Immunohistochemistry for angiogenesis marker was conducted as previously described [20], with exception the CD31 antibody was purchased from Histonova (Clone SZ31).

### Cleaved PARP fragment quantification

Levels of cleaved PARP product were semi-quantified from autoradiography film by densitometry using ImageQuant TL software (GE Healthcare Life Sciences). Quantification of soluble cleaved PARP levels in xenograft tumor lysates was determined using the MSD cleaved PARP (Asp214) assay whole cell lysate kit (Meso Scale Discovery, Gaithersburg, MD). Each tumor lysate was assayed in triplicate using 100 µl of lysate at 0.2 µg/µl in 96-well assay plates.

### Affymetrics, multiplex gene expression quantification and CCLE

RNA expression levels for select genes *MCL1*, *BCL-xL (BCL2L1*), *BCL2*, *GAPDH*, and *TUBA1B (α-tubulin)* from dinaciclib-treated cells were quantified by a microsphere-based multiplex branched DNA assay (QuantiGene 2.0 Multiplex assay, Panomics/Affymetrix, Fremont, CA) using Plex Set 11837. Samples were analyzed using a Bio-Plex 200 System Array reader with Luminex 100 xMAP technology, and the data were acquired using Bio-Plex Data Manager version 5.0 (Bio-Rad, Hercules, CA). Cells were cultured and drug treated in 10 cm dishes, detached by trypsinization and 150,000 cells were processed according to the manufacture's protocol. mRNA data were normalized to the geometric mean of *TUBA1B* and *GAPDH* mRNA levels.

Reported values for *MCL1* and *BCL2L1* gene expression were obtained by processing Affymetrix CEL files for CCLE cell lines with RefRMA algorithm using default settings with subsequent log10 transformation applied to generated probeset intensities. CEL files (GEO accession: GSE36133) were downloaded from the CCLE project website at the Broad Institute: http://www.broadinstitute.org/ccle [21]. RefRMA algorithm implementation was downloaded from the Affymetrix website (http://www.affymetrix.com/partners_programs/programs/developer/tools/powertools.affx) and standard version of U133 2.0 + CDF file was used (http://www.affymetrix.com/Auth/support/downloads/library_files/hgu133plus2_libraryfile.zip). For both genes we used REFSEQ-based probesets: 200798_x_at (for *MCL1*) and 206665_s_at (for *BCL2L1*). *MCL1* gene copy number data was obtained from a pending 2014 CCLE release (Gregory Kryukov, personal communication).

### Cell viability assay, cell counting, caspase-3/7 activity assay and cell cycle analysis

Cell viability was determined by measuring ATP using the CellTiter-Glo Luminescent Cell Viability Assay (Promega, Madison, WI) according to the manufacturer's instructions. Drug potency was calculated as the ratio of relative light units (RLUs) in compound treated wells over DMSO-treated control wells and expressed as % DMSO control. Cell number and trypan blue dye exclusion viability was assessed using a Vi-CELL Cell Viability Analyzer (Beckman Coulter, Indianapolis, IN). Caspase-3 and -7 activities were measured using the Caspase-Glo 3/7 Assay (Promega) in a 96-well plate format according to the manufacturer's instructions. Cell cycle analysis was assessed by propidium iodide (PI) staining. The DNA content profile was determined on a BD FACSCalibur flow cytometer and data were analyzed using FlowJo software.

### *MCL1* siRNA knockdown and rescue

NCI-H23 cells were infected with control-RFP or *MCL1* lentivirus at MOI  = 1 (Open Biosystems/Thermo # OHS5833, #OHS5899-202617926) in the presence of 4 ug/ml polybrene (Sigma). MCL1 overexpression was confirmed by immunoblot 3 days after selection with blasticidin (InvivoGen, 5 ug/ml). Following expansion under selection, cells were reverse transfected with control (Dharmacon #D-00180-10-05) or *MCL1* siRNA (Dharmacon #J-004501-17-0002) targeting the 3′ UTR of *MCL1* which is not present in the *MCL1* overexpression construct. Following 24 hr of knockdown, cell viability was measured by CellTiter-Glo (Promega) and MCL1 expression was examined by immunoblot.

### Bliss independence calculations

Bliss independence (synergy) calculations were determined by dose-matrix combination response [22]. Cells were treated for 18 hr with an 8-point dose-dilution series matrix combination of dinaciclib (400–3.1 nM) and navitoclax (4000–31.3 nM), as well as treating cells with the same dilution series of each drug alone. The dilution series consisted of eight, 2-fold dilutions. The percent cell viability was determined relative to DMSO-treated cells using CellTiter-Glo.

### Ethics Statement

All animal studies were performed according to the protocols approved by each institution's Institutional Animal Care and Use Committee, following the guidance of the Association for Assessment and Accreditation of Laboratory Animal Care International (AAALAC).

### *In vivo* efficacy and PD studies

Dinaciclib was formulated in 20% hydroxypropyl beta-cyclodextrin (HPBCD, Fisher # NC9686303) in de-ionized (DI) water (vehicle). 80 mg dinaciclib was added to 10 mL room-temperature (RT) sterile-filtered vehicle and dissolved using a magnetic stirring bar at RT for at least 12 hours. The formulation was stored at 5°C, used within 7 days and warmed to RT and vortexed for 3 seconds before i.p. administration. Formulation and administration of KDR inhibitor compound B was performed as previously described [19]. Xenograft NCI-H23 and COLO-320DM pharmacodynamic tumor studies (PD study) were conducted by MRL-Boston; COLO-320DM efficacy study was conducted by WuxiApptec (Shanghai, China); A2780, 22Rv1, JIMT-1, MDA-MB-231 and PC3 tumor studies were conducted by Piedmont Research Center (Morrisville, NC). Implantation consisted of 1×10^7^ A2780 cells in PBS, 5×10^6^ MDA-MB-231 cells in PBS, ∼1 mm^3^ PC3 tumor fragments in female athymic nude mice (Crl:NU(NCr)-*Foxn1nu*, Charles River Laboratories); 5×10^6^ NCI-H23 in 50% Matrigel (BD Biosciences), 5×10^6^ COLO-320DM cells in 50% Matrigel (PD study only), 1×10^7^ JIMT-1 cells in 50% Matrigel in female SCID mice (Fox Chase SCID, C.B-17/Icr-*Prkdcscid*, Charles River Laboratories); 1×10^7^ 22Rv1 cells in 50% Matrigel in the flanks of male athymic nude mice (*nu/nu*, Harlan); 1×10^7^ COLO-320DM cells in PBS (efficacy study only) in female BALB/c nude mice (Sino-British SIPPR/BK Lab. Animal Co. Ltd). For xenograft efficacy studies, 40 mg/kg dinaciclib or control vehicle were dosed intraperitoneal (i.p.) twice weekly schedule (days 1 and 4) for NCI-H23 and q4d for all others. Groups consisted of 10 animals each, except for JIMT-1 (dinaciclib group only) consisted of 8 animals each. The difference between the mean values of tumor size at end of study in dinaciclib-treated and vehicle groups was analyzed for significance by a t-test. All data were analyzed using Microsoft Excel and GraphPad Prism software. *p*<0.05 was considered to be statistically significant. Tumor growth inhibition % (TGI %) was calculated according to the following equation: TGI (%)  =  [−100 × (T_1_-T_0_)/(C_1_-C_0_)] + 100, wherein C_1_ is the mean tumor volume of control mice at time t; T_1_ is the mean tumor volume of treated mice at time t; C_0_ is the mean tumor volume of control mice at time 0; T_0_ is the mean tumor volume of treated mice at time 0. C_1_ and T_1_ were measured at the end of study date and C_0_ and T_0_ were measured at the treatment start date. For PD analysis, tumor samples were collected 6 hr post dosing from animals in two sampling groups (n = 5) that received one dose of control vehicle or dinaciclib at 40 mg/kg. Tumor samples were either snap frozen in liquid nitrogen and stored at −80°C or preserved in 10% neutral buffered formalin for 24 hours then stored in 70% ethanol at ambient temperature. Blood concentrations of dinaciclib were determined by protein precipitation followed by liquid chromatography–tandem mass spectrometry. Pharmacokinetics blood samples were collected by tail-clip and 10 µL of whole blood mixed with 30 µL of 0.1 M sodium citrate were used for analysis. Blood samples obtained from dosed animals were prepared for analysis by means of a single step protein precipitation technique by adding 200 µL of acetonitrile containing internal stardard to the aliquots of individual subject samples. Samples were mixed by vortex for homogeneity and then subjected to centrifugation at 3500 rpm for 10 min. The supernatant (200 µL) was collected and a volume of 5 µL was injected into the LC-MS/MS for analysis and concentrations in unknown samples were calculated from the best-fit equation of a dinaciclib stanadard curve prepared in blank mice blood matrix.

## Results

### *MCL1:BCL-xL* mRNA ratio and *MCL1* copy number correlate with dinaciclib sensitivity in solid tumor cell lines

**Figure 1:**
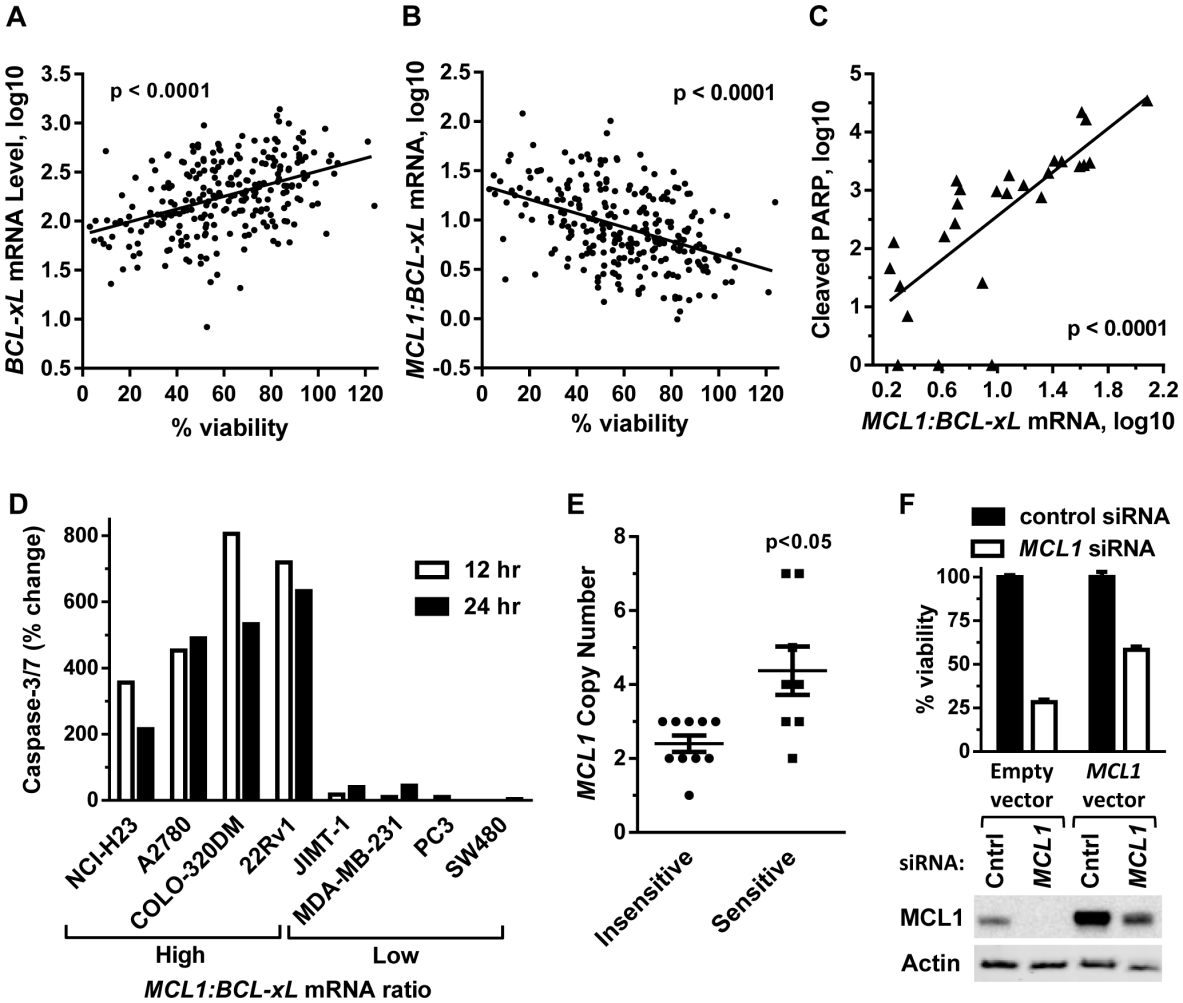
Sensitivity to short-term dinaciclib treatment correlates with *BCL-xL* and *MCL1* levels. (A) *BCL-xL* mRNA level positively correlated with viability of 254 solid tumor cell lines treated 24 hr with dinaciclib (100 nM). (B) The *MCL1:BCL-xL* mRNA ratio negatively correlated with viability in the same dinaciclib-treated cell line panel as (A). (C) Effect of 8 hr dinaciclib (100 nM) treatment on apoptosis induction as measured by cleaved PARP fragment (y axis) against the *MCL1:BCL-xL* mRNA ratio (x axis). Data were measured from 27 cell lines. The *MCL1:BCL-xL* mRNA ratios and relative cleaved PARP fragment values for these cell lines are listed in Supplemental Table S1. Relative cleaved PARP fragments values <1 were adjusted to 1. *MCL1* and *BCL-xL* expression values were obtained from the Broad Institute CCLE. (D) Percent change in activated caspase-3/7 levels after 12 hr (empty bars) and 24 hr (filled bars) of dinaciclib (100 nM) treatment in four high *MCL1:BCL-xL* mRNA ratio cell lines (COLO 320DM, 22Rv1, A2780, NCI-H23) and four low *MCL1:BCL-xL* mRNA ratio cell lines (MDA-MB-231, JIMT-1, SW480, PC3). (E) Effect of *MCL1* copy number on viability after 24 hr, 100 nM dinaciclib treatment in 18 cell lines. Insensitive cell lines (circles) exhibited ≥70% cell viability and sensitive cell lines (squares) exhibited <30% cell viability after dinaciclib treatment. (F) *MCL1* rescue of siRNA knockdown in NCI-H23 cells. Top, percent cell viability remaining in cells transfected with empty vector control or vector expressing *MCL1* lacking 3′ UTR, followed by 24 hr transfection with control siRNA (filled bar) or *MCL1* siRNA targeting 3′ UTR of endogenous *MCL1* (empty bar). Bottom, MCL1 immunoblot of NCI-H23 cells described in top panel. Cntrl  =  Control.

To begin understanding the mechanism(s) associated with dinaciclib sensitivity using treatment times more relevant to clinical exposure, gene expression of >250 solid tumor cell lines were correlated to cell viability following a 24 hour (hr) dinaciclib treatment. Dinaciclib sensitivity positively correlated with expression levels of antiapoptotic BCL2-family member *BCL-xL (BCL2L1)*, such that low *BCL-xL* expression correlated with low cell viability remaining after dinaciclib treatment (p<0.0001, Figure 1A). In addition to this correlation being observed across solid tumor cell lines of multiple indications, the correlation was also observed within 8-of-12 individual cancer types where ≥6 cell lines were available for analysis (Table S2). This finding is consistent with a recent report demonstrating a correlation between cell viability and *BCL-xL* levels after treatment with pan-CDK inhibitor flavopiridol [23]. Given dinaciclib's ability to downregulate the antiapoptotic protein MCL1 [10], [11], we also observed that the *MCL1:BCL-xL* mRNA ratio negatively correlated with dinaciclib sensitivity (high ratio lines exhibited lower viability), consistent with the functional redundancy of these two antiapoptotic proteins (p<0.0001, Figure 1B). High *MCL1:BCL-xL* mRNA ratio also positively correlated with dinaciclib-induced apoptosis as measured by the apoptotic marker cleaved poly (ADP-ribose) polymerase 1 (PARP) (p<0.0001) (Figures 1C and Table S1) and caspase-3/7 activation (Figure 1D).

Immunoblot analysis of 13 human cancer cell lines analyzed in figure 1C, representing a mixture of solid tumor types with a broad range of *MCL1:BCL-xL* mRNA ratios, confirmed that MCL1 and BCL-xL protein levels correlated with the reported *MCL1* and *BCL-xL* mRNA levels (Figure S1). Treatment of these cells with a clinically achievable concentration of dinaciclib for 8 hr resulted in complete target engagement as measured by CDK9 phosphorylation of Ser2 within RNAP II CTD, referred to as RNAP II P-Ser2. MCL1 levels were reduced in all cell lines after dinaciclib treatment while BCL-xL and BCL2 protein levels were unchanged. Dinaciclib induced apoptosis, as measured by cleaved-PARP, was greatest in the higher *MCL1:BCL-xL* ratio cell lines, which negatively correlated with cell viability (Figure S1).

In agreement with the *MCL1:BCL-xL* mRNA ratio being predictive of dinaciclib sensitivity, cells which were *MCL1* amplified were also more sensitive to dinaciclib relative to *MCL1* non-amplified cells (Figure 1E, Table S3). In this same panel of cells, 3-of-7 dinaciclib-sensitive and *MCL1* amplified cell lines were shown to be *MCL1*-dependent as assessed by *MCL1* siRNA knockdown (Table S3). The non-small cell lung carcinoma (NSCLC) cell line NCI-H23 was highly sensitive to *MCL1* siRNA knockdown (Table S3) in agreement with previous findings [24]. *MCL1* knockdown could be partially rescued by exogenous *MCL1* expression (Figure 1F). As *MCL1* amplification and high *MCL1:BCL-xL* ratio were associated with dinaciclib sensitivity, these data suggest that the antiapoptotic protein MCL1 is important to dinaciclib's mechanism of action and a relevant predictive biomarker *in vitro*.

### Dinaciclib *in vitro* response correlates with triptolide which is known to be associated with MCL1-dependency

**Figure 2:**
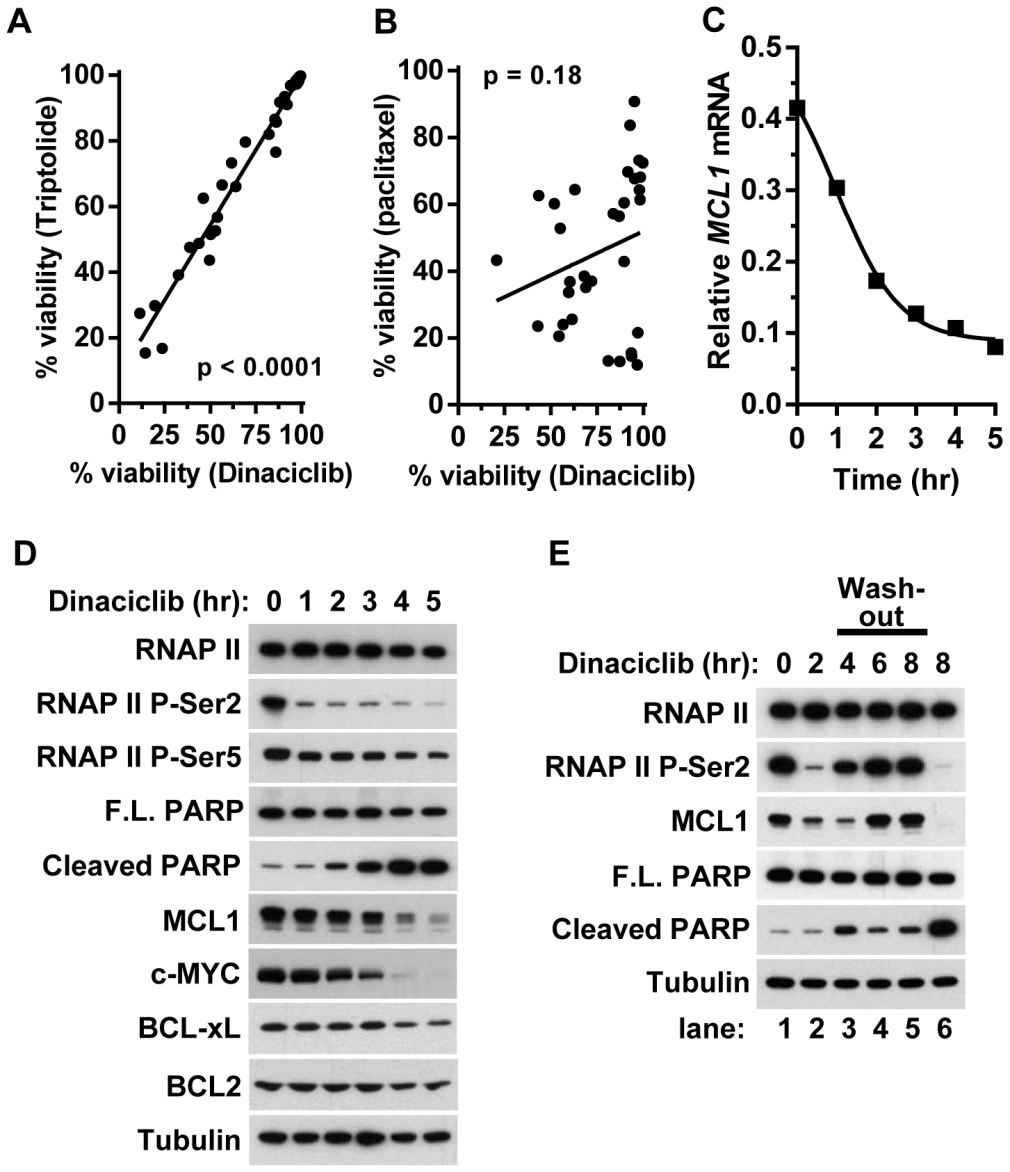
Dinaciclib functions as a transcriptional repressor requiring >2 hr exposure to induce apoptosis. (A) Effect of dinaciclib (100 nM) compared to transcriptional repressor triptolide (3 µM) in 33 ovarian cell lines after a 24 hr treatment. (B) Effect of dinaciclib (100 nM) compared to paclitaxel (3 µM) in 33 ovarian cell lines after a 24 hr treatment. (C) Dinaciclib (100 nM) downregulates *MCL1* mRNA expression levels in A2780 cells during a 5 hr treatment. Expression level was normalized to the geometric mean of α*-tubulin* and *GAPDH* mRNA levels. (D) Immunoblot analysis of A2780 cells during the 5 hr time-course in (C). (E) Immunoblot analysis of A2780 cells treated with dinaciclib (100 nM) for 0, 2 or 8 hr (lanes 1, 2 and 6). After 2 hr treatment, dinaciclib was washed-out and cells were analyzed at subsequent 2 hr intervals with the cumulative times from t = 0 as indicated (lanes 3, 4 and 5).

To determine if dinaciclib cell-killing is associated with agents previously shown to correlate with BCL-xL expression and MCL1 dependence, we compared the cell viability response between dinaciclib and the RNA polymerase inhibitor triptolide in 33 ovarian cell lines. Triptolide was previously shown to function as an inhibitor of transcription [25] and the viability of triptolide treated cells correlated with *BCL-xL* expression levels and *MCL1* copy number [23]. Here we observed that cells sensitive to dinaciclib showed a positive correlation (p<0.001) to triptolide in a 24 hr viability assay (Figure 2A), which was not observed with the anti-mitotic agent paclitaxel used as comparison to an alternative mechanism (p = 0.18) (Figure 2B). In agreement with these observations and previous findings with other CDK9 inhibitors [12]–[14], dinaciclib caused a rapid decrease in *MCL1* mRNA levels within the first 2 hr of treatment that plateaued at 4–5 hr in the A2780 ovarian cancer cell line (Figure 2C). This decrease in *MCL1* mRNA coincided with the rapid loss of the CDK9 phosphorylation site RNAP II P-Ser2 (Figure 2D). Shorter time-course treatments showed that RNAP II P-Ser2 levels began to decrease within 15 min of dinaciclib addition (data not shown). Consistent with kinase selectivity data, dinaciclib treatment only caused a moderate reduction of the CDK7 phosphorylation site RNAP II P-Ser5 [26]. The kinetics of *MCL1* mRNA loss coincided with decreased MCL1 protein levels, with near complete loss after 5 hr (Figures 2C and 2D). Similar results were observed with c-MYC which also has a short half-life of approximately 25 minutes [27]. While dinaciclib treatment also decreased mRNA levels of antiapoptotic family members *BCL2* and *BCL-xL* (Figure S2), this had relatively minimal impact on BCL2 and BCL-xL protein levels (Figure 2C), which are known to have much longer half-lives than MCL1 [28], [29]. The decrease of MCL1 coincided with a temporal increase in cleaved PARP fragment. The kinetic increase in the cleaved PARP fragment measured using the caspase-dependent neoepitope antibody PARP (Asp214) is more apparent than the decrease in full length PARP and therefore the focus of analysis in subsequent experiments. Similar kinetics in *MCL1* mRNA, protein responses, and apoptosis induction were observed with NCI-H23 cells (Figure S2).

While 4 hr dinaciclib treatment results in robust induction of apoptosis as measured by cleaved-PARP (Figure 2D), we were interested to test if shorter treatment in solid tumor cell lines could induce equivalent levels of apoptotic induction. As such, we treated A2780 cells for 2 hr with dinaciclib followed by compound wash-out and compared the response to 8 hr of continuous treatment (Figure 2E). While 2 hr of treatment diminished RNAP II P-Ser2 and began to diminish MCL1 protein, both biomarkers rapidly returned to baseline levels following removal of dinaciclib and resulted in little to no induction of apoptosis after 6 hr of recovery compared to 8 hr of continuous treatment (Figure 2E). Similar results were observed in a dinaciclib wash-out study using NCI-H23 cells (Figure S2). Shorter time-course experiments showed recovery of RNAP II Ser2 phosphorylation beginning within 30 min of dinaciclib removal (data not shown). These results suggest that a sustained inhibition of RNAP II greater than 2 hr is required to decrease MCL1 to levels needed for commiting cells to apoptotic induction observed with 4-to-8 hr of dinaciclib treatment *in vitro*.

### Dinaciclib and navitoclax-induced cytotoxicity are inversely related and the combination is synergistic

**Figure 3:**
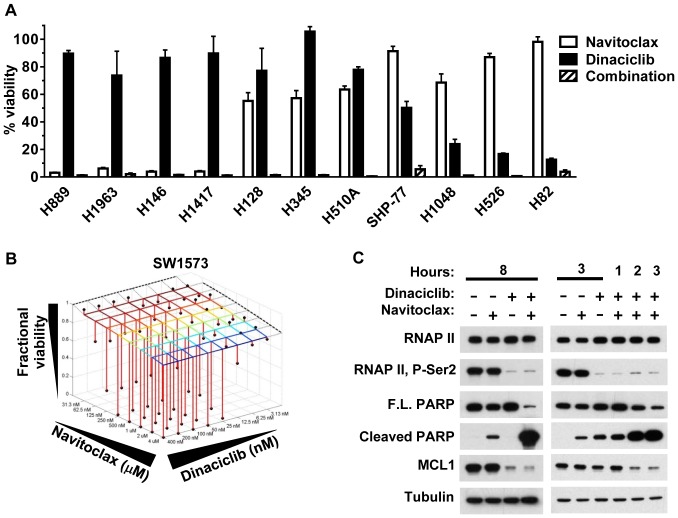
Dinaciclib and navitoclax have an inverse cell-killing relationship and the combination is synergistic. (A) Effects of 24 hr treatment of 1 µM navitoclax (open bars), 100 nM dinaciclib (filled bars) and the combination at the respective concentrations (hatched bars) on cell viability in 11 SCLC cell lines. (B) Bliss synergy analysis graph of the expected fractional cell viability response to the combination of dinaciclib and navitoclax at specified concentrations (grid intersections) compared to the observed (black balls) fractional cell viability response following 18 hr treatment using an 8×8 dose escalation matrix. The dotted lines correspond to the fractional viability of dinaciclib (left, rear) and navitoclax (right, rear) treatment alone at the specified concentrations. (C) Immunoblot analysis of total protein lysates from SW1573 cells treated for the indicated times with 1 µM navitoclax, 100 nM dinaciclib or the combination.

To further investigate dinaciclib's functional effect on the intrinsic apoptosis pathway, single-agent and combination treatments of dinaciclib and navitoclax were examined in a panel of 11 small cell lung cancer (SCLC) cell lines. Navitoclax (ABT-263) is a small-molecule inhibitor of antiapoptotic BCL2 related proteins BCL2, BCL-xL and BCL-w, but not MCL1 [30], [31]. An earlier report characterized a navitoclax-related inhibitor, ABT-737, which demonstrated an inverse relationship between ABT-737-induced cytotoxicity and MCL levels in the majority of the cell lines tested [32]. We observed a similar response of these cells to that previously described for ABT-737 and navitoclax [31], [32] and that dinaciclib showed an opposing response profile to ABT-737, with four of the most navitoclax-sensitive cell lines being dinaciclib-resistant and the three most dinaciclib-sensitive cell lines being navitoclax-resistant (Figure 3A). We hypothesized that the ability of navitoclax to block BCL2, BCL-xL and BCL-w function and dinaciclib to diminish the MCL1 would result in synergistic cell-killing. In agreement with this, all 11 SCLC cell lines were highly sensitive to the dinaciclib and navitoclax combination at concentrations where at least one of these agents was ineffective (<35% cell viability loss) (Figure 3A). To further interrogate whether the combination effect of dinaciclib and navitoclax were synergistic or additive, Bliss independence analysis was performed on five additional cell lines using a 8×8 concentration matrix centered around 50 nM for dinaciclib and 500 nM for navitoclax. Four *MCL1:BCL-xL* low ratio cell lines (SW1573, PC-3, SW480 and MDA-MB-231), which were insensitive to single-agent dinaciclib and navitoclax, and one *MCL:BCL-xL* high ratio, dinaciclib-sensitive control cell line (NCI-H23) line were treated with this combination matrix for 18 hr and then assayed for viability. The dinaciclib-navitoclax combination in the four *MCL1:BCL-xL* low ratio cell lines exhibited strong synergy (Bliss >0.1) in reducing cell viability (Table 1, Figure 3B and Figure S3). These results are consistent with the four *MCL1:BCL-xL* low ratio cell lines having functionally redundant antiapoptotic mechanisms that are independently inhibited by dinaciclib (MCL1) and navitoclax (BCL-xL, BCL-2, BCL-w). The neutral combination effect in NCI-H23 cells is consistent with this cell line being exclusively dependent upon MCL1 and as previously reported (Figure 1F and [33]). Western blot analysis of SW1573 cell lysates further demonstrated dinaciclib selectivity for RNAP II P-Ser2 inhibition and MCL1 downregulation as compared to single-agent navitoclax, and minimal single-agent but robust dinaciclib-navitoclax combination induced apoptosis (Figure 3C). Induction of apoptosis from the dinaciclib and navitoclax combination was more rapid than single agent treatment, occurring within 2 hr, and consistent with repression of all antiapoptotic BCL2 family members (Figure 3C).

**Table 1:** Summary of Bliss scores and sensitivity to dinaciclib, navitoclax and combination treatment.

	Dinaciclib plus Navitoclax	Dinaciclib only	Navitoclax only	Dinaciclib plus Navitoclax	*Mcl-1: Bcl-xL*	*MCL1*	*BCL-xL*
Cell Line	vBliss^1^	% viability^2^	% viability^2^	% viability^2^	mRNA ratio^3^	mRNA^3^	mRNA^3^
SW1573	0.42	112	92	1	0.62	3.00	2.39
PC-3	0.11	92	103	54	0.28	3.05	2.77
SW480	0.27	109	93	4	0.22	2.95	2.73
MDA-MB-231	0.21	93	83	9	0.35	3.14	2.79
NCI-H23	0.03	52	88	39	1.64	3.40	1.75

1 Synergy analysis after 18 hr treatment. vBliss: synergy (>0.1), additive (0.1–0.05). neutral (<0.05-0), antagonistic (<0).

2 Percent viability after 18 hour treatment with 100 nM dinaciclib, 1 µM navitoclax or the combination.

3 Information source. Cancer Cell Line Encyclopedia converted from log 2 to log10.

**Figure 4:**
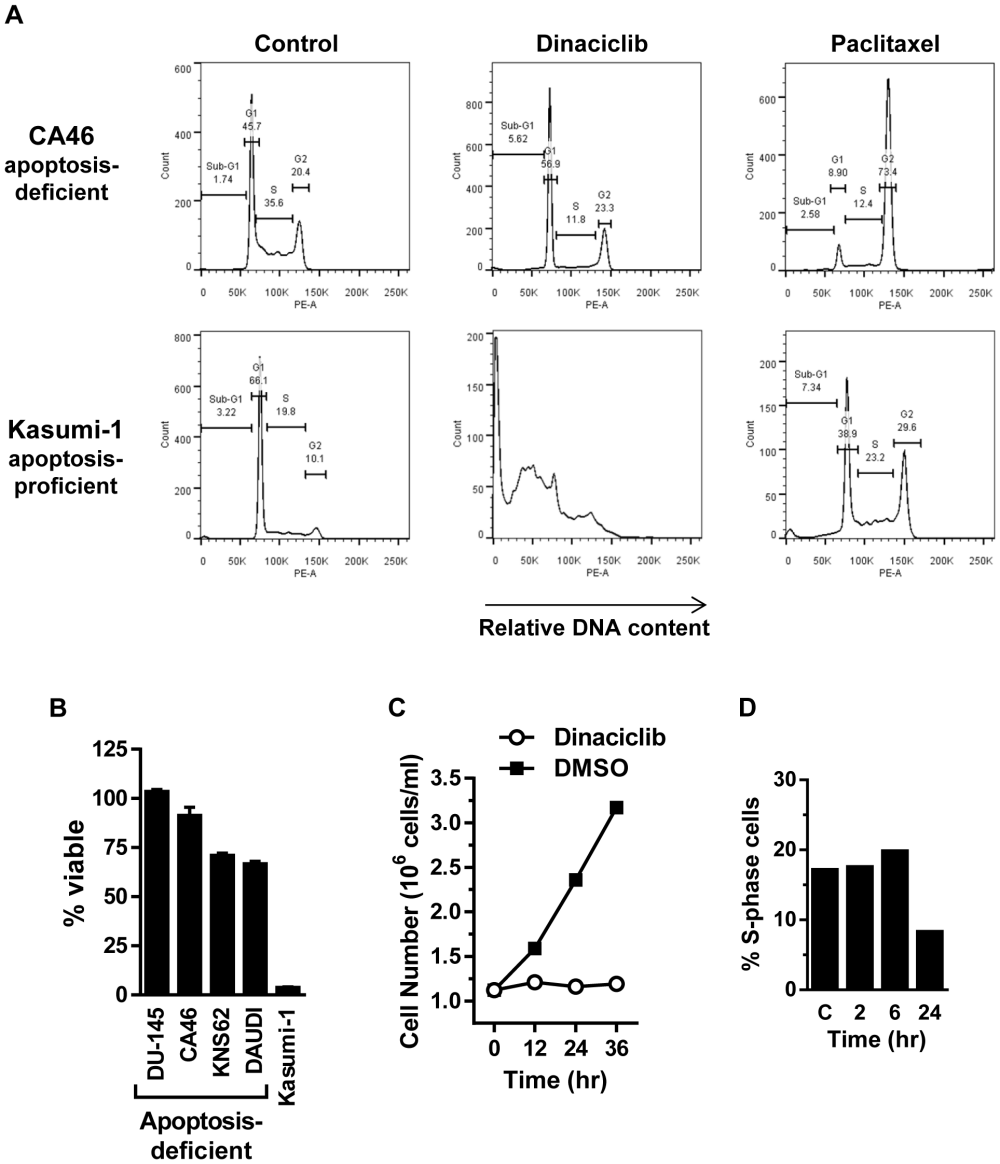
Cell cycle analysis of dinaciclib in apoptotic-proficient and apoptotic-deficient cell lines. (A) Apoptotic-deficient CA46 cells and apoptotic-proficient Kasumi-1 cells were treated for 24 hr with dinaciclib (100 nM) or mitotic inhibitor paclitaxel (3 µM). DNA content was analyzed by propidium iodide staining and flow cytometry to determine cell cycle effects. Bars used to calculate the percent of cells in sub-G1 (apoptotic) or G1, S, G2 cell cycle phases are shown. (B) Percent cell viability remaining in apoptotic-deficient (DU-145, CA46, KNS62, DAUDI) and apoptotic-proficient (Kasumi-1) cells after 24 hr dinaciclib (100 nM) treatment. (C) Cell proliferation of apoptotic-deficient CA46 cells during 36 hr dinaciclib (100 nM) (open circles) or DMSO-control (filled squares) treatment. (D) Apoptotic-deficient CA46 cell line treated 2 and 6 hr with dinaciclib (100 nM) followed by drug wash-out and 24 hr recovery. DMSO-treated control cells and 24 hr continuous dinaciclib (100 nM) treated cells are represented by C and 24 hr, respectively. DNA content was analyzed by propidium iodide staining and flow cytometry and the percent S-phase cell population is shown. The failure of the S-phase peak to decrease after a transient 2 and 6 hr dinaciclib treatment indicates rapid recovery of CDK1 and 2 functions.

Additional evidence of dinaciclib working through an apoptotic-inducing mechanism was further supported by the requirement for downstream pro-apoptotic effectors BAX and BAK. CA46, DU-145, Daudi cells, which are reported to be defective for pro-apoptotic proteins BAX/BAK [34]–[37] and KNS62 cells which harbor a BAX (G67R) inactivating mutation [37], as observed in the Catalogue of Somatic Mutations in Cancer (COSMIC) database, did not show induction of apoptosis, assessed by the failure to detect sub-G1 DNA content cells by flow cytometry, as compared to apoptotic-proficient Kasumi-1 cells [38] (Figure 4A and Table S4). The response to paclitaxel demonstrated that the cells were actively dividing and responsive to anti-mitotic agents as measured by flow cytometry. Furthermore, the four apoptotic-defective cell lines retained >70% cell viability after 24 hr dinaciclib treatment while <10% viable cells were detected in Kasumi-1 cells (Figure 4B). In agreement with these findings, BAX/BAK-deficient CA46 cells failed to induce apoptosis, assessed by cleaved PARP, and retained >90% cell viability following an 18 hr treatment of the dinaciclib and navitoclax drug combination (data not shown). Collectively, these data demonstrate that a major mechanism of dinaciclib's effect *in vitro* is transcriptional repression resulting in decreased abundance of short half-life MCL1. Subsequent induction of apoptosis is dependent on the levels of compensatory antiapoptotic family members, which correlates with the *MCL1:BCL-xL* mRNA ratio or *MCL1* copy number.

### Apoptotic BAX/BAK-defective cell line allows observation that dinaciclib induces a G1 and G2 cell cycle block

The inability of dinaciclib to induce apoptosis in BAX/BAK-defective cells provides a means to examine the degree to which short-term dinaciclib induced cell cycle arrest contributes to overall cell viability readouts. Dinaciclib had an immediate antiproliferative effect on apoptotic-deficient CA46 cells that was maintained throughout a 36 hour treatment period, a time period in which the cell number of the DMSO control culture nearly tripled (Figure 4C). During this time there was only a minor reduction in cell viability as measured by trypan blue dye exclusion (97% viable at t = 0 versus 93% viable at t = 36 hours) and as described above, little to no evidence of apoptosis. Flow cytometry analysis of the four apoptotic-defective cell lines (CA46, DU-145, KNS62 and Daudi) demonstrated dinaciclib's ability to inhibit cell cycle progression at both G1/S and G2/M with 24 hr of treatment, as reflected by minor changes in the corresponding G1 and G2/M peaks and 2–3 fold decrease of S-phase cells (Table S4). The cell cycle blocks impacted overall cell viability by <30% in the four apoptosis-deficient cell lines as measured by cellular ATP content (Figure 4B). Additionally, using CA46 cells, a transient 2 or 6 hr dinaciclib treatment followed by 24 hr recovery showed no decrease in the S-phase peak (Figure 4D), consistent with the rapid recovery of CDK activity upon dinaciclib wash-out shown above (Figure 2D). In total, these data show that the contribution of cell division arrest to overall dinaciclib-induced cell viability loss is negligible during short-term dinaciclib treatments. Furthermore, dinaciclib exposure times of ≤6 hr are fully reversible and have little overall impact with regards to cell cycle inhibition, while dinaciclib-induced apoptosis has sustained and permanent impact on cell viability.

### Pharmacokinetics and pharmacodynamics of dinaciclib indicate short-term *in vivo* exposure is sufficient to induce tumor apoptosis

Clinical pharmacokinetic (PK) analysis of patient plasma samples obtained during and after 2 hr dinaciclib IV infusion showed that dinaciclib exhibits rapid elimination 6 hr post-infusion stop, demonstrating a mean terminal half-life (t1/2) of ∼3.3 hr at the RP2D of 14 mg/m^2^ [5], [6], [18]. At the 14 mg/m^2^ dose, dinaciclib mean plasma concentrations were ∼2000, 200 and 100 nM at 2, 4 and 6 hr, respectively, after starting the 2 hr infusion [18]. Comparing human PK to mouse PK data, dinaciclib exhibits a more rapid clearance in mice with a t1/2 of 0.75 hr using a 40 mg/kg, i.p. dose that is routinely used for mouse tumor xenograft studies (Figure S4). Dinaciclib plasma levels in mouse models peak ∼15 min post-injection (∼5 µM) and are detectable at 2, 4, 6 hours (∼330, 65, 30 nM respectively). Thus, dinaciclib has a shorter exposure time in mice than in human with this dosing schedule.

**Figure 5:**
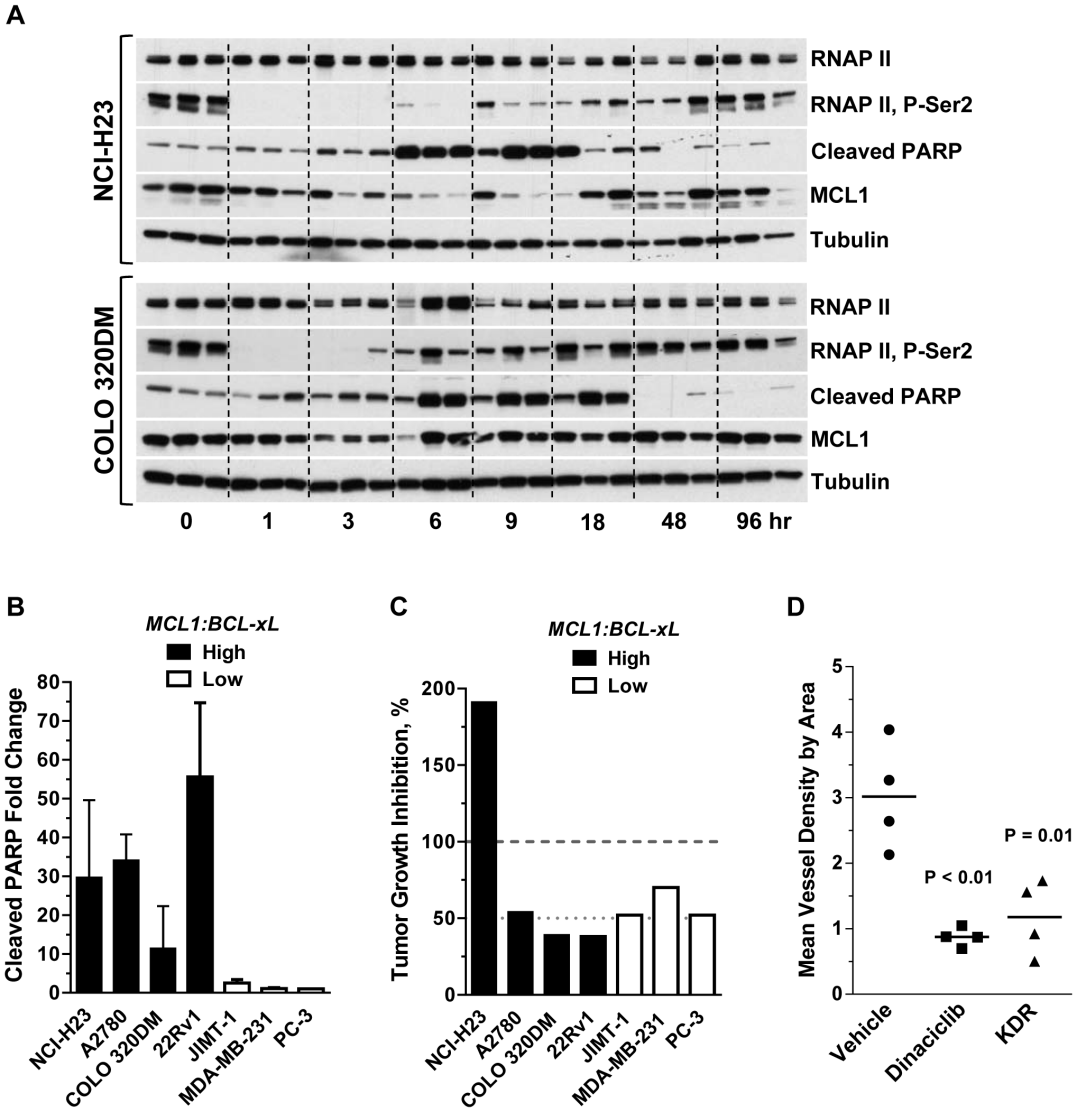
Dinaciclib exhibits apoptotic-induction, tumor efficacy and antiangiogenic activity in human tumor xenograft models. (A) Immunoblot analysis of lysates prepared from NCI-H23 and COLO 320DM xenograft tumors resected at 1, 3, 6, 9, 18, 48 and 96 hr after a single 40 mg/kg, i.p. dinaciclib injection. Time 0 is a 1 hr vehicle treatment. Represented are three tumors from three animals per time-point. α-tubulin is included as a loading control. Quantification of the cleaved-PARP fragment in these lysates is shown in supplemental Figure S4. (B) Quantification of cleaved-PARP fragment in lysates prepared from tumors resected 6 hr post-administration of vehicle or a single dose of dinaciclib at 40 mg/kg, i.p. Xenograft models designated as *MCL11:BCL-xL* high (filled bars) or low (open bars) mRNA ratio as defined in Figure S1 and Table S1. Fold change was determined from the mean of 3–5 dinaciclib-treated and 3–5 vehicle-treated tumors for each model. The mean and standard deviation are shown. (C) Efficacy of dinaciclib in 7 human xenograft models. Xenograft models designated as *MCL11:BCL-xL* high (filled bars) or low (open bars) mRNA ratio are indicated. Dinaciclib was given at 40 mg/kg, i.p., twice-weekly or 4qd. %TGI was measured at the end of the dosing period (n = 10 mice per group, except n = 8 mice in the JIMT-1 dinaciclib group). Tumor growth rates over the course of the study and endpoints are shown in Supplemental Figure S6. All dinaciclib-treated groups had mean tumor volumes that were significantly smaller than vehicle-treated groups at the end of study (p<0.05). (D) Antiangiogenic effect of dinaciclib relative to KDR inhibitor in A2780 xenograft tumors. Dinaciclib was given at 40 mg/kg, i.p., days 1, 4, 7 and KDR inhibitor was given at 10 mg/kg, po, days 1–7. Tumors were resected 2–4 hours after the last drug treatment. KDR-inhibitor compound B [19] was utilized as a positive control in these studies. Mean vessel density by area  =  % of endothelial cells divided by the total tissue area of interest [20].

Pharmacodynamic (PD) analysis of a single 40 mg/kg i.p. dinaciclib dose was evaluated in NCI-H23 and COLO 320DM xenograft tumors as these cells showed apoptotic responses following dinaciclib treatments *in vitro* (Figures 1D and S1 and Table S1). Immunoblot analysis showed rapid elimination of RNAP II P-Ser2 1 hr post administration, MCL1 downregulation at 3 hr, and apoptosis induction (cleaved-PARP) beginning at 6 hr that was sustained for ≥12 hr (Figure 5A and S4). Similar to dinaciclib *in vitro* wash-out experiments (Figure 2D), recovery of RNAP II P-Ser2 and MCL1 levels coincided with clearance of dinaciclib, having recovered to baseline 6 hours post-administration, though recovery to base-line levels was delayed ∼3 hours in NCI-H23 tumors compared to COLO 320DM. Thus the kinetics of CDK9 target engagement and apoptosis induction *in vivo* translates to those observed *in vitro* for dinaciclib.

### The *MCL1:BCL-xL* mRNA ratio is predictive of dinaciclib-induced apoptosis *in vivo*

To investigate the potential translation of the *MCL1:BCL-xL* mRNA ratio as a predicative biomarker of dinaciclib-induced apoptosis to the *in vivo* setting, four *MCL1:BCL-xL* high ratio (NCI-H23, A2780, COLO-320DM, 22Rv1) and three low ratio (JIMT-1, MDA-MB-231, PC-3) xenograft tumor models were selected (see Figure S1) for dinaciclib pharmacodynamic evaluation. Based on dinaciclib's PK properties in mice and kinetics of apoptosis induction described above, tumor lysates from these seven xenograft models were evaluated for apoptosis induction 6 hr after a single dinaciclib 40 mg/ml i.p. injection. Consistent with our *in vitro* findings, dinaciclib-induced apoptosis was observed only in the four *MCL1:BCL-xL* mRNA high ratio tumor models (Figure 5B). Strikingly, dinaciclib treatment induced an 11-to-56 fold increase in cleaved PARP fragment levels in tumors from the four *MCL1:BCL-xL* high ratio xenograft models compared to a ≤2.5-fold increase of cleaved PARP in the tumors from the low ratio xenograft models (Figure 5B). Evidence of CDK9 inhibition based on RNAP II P-Ser2 and MCL1 downregulation was apparent across all 7 xenograft tumor models (Figure S5) even though measuring RNAP II P-Ser2 at the 6 hr time point used for this study, focusing on apoptotic response, was slightly late for those early markers based on kinetic studies described above (Figure 5A).

To evaluate whether the dinaciclib-induced apoptosis response in tumors translated to an overall anti-tumor growth effect, efficacy studies were conducted in these same seven tumor xenograft models. Dinaciclib caused significant tumor regression (%TGI  = 191%) in the *MCL1:BCL-xL* high mRNA ratio and MCL1-dependent NCI-H23 xenograft model (Figures 5C and S6). Amongst the six other xenograft tumor models, dinaciclib exhibited low to moderate (38–70% TGI) antitumor effects. These data demonstrate that while dinaciclib-induced intra-tumoral apoptosis correlated with the *MCL1:BCL-xL* mRNA ratio, the cumulative antitumor growth effect was inconsistent with this biomarker when dinaciclib is given at a 40 mg/kg, q4d dose schedule. Additionally, the observation that the three non-apoptosis responsive tumor xenografts (JIMT-1, MDA-MB-231 and PC3) exhibited >50% TGI suggests that additional tumor cell intrinsic or microenviroment mechanisms in the *in vivo* setting may come into play which impact dinaciclib's antitumor effect.

To begin assessing additional microenvironment or tumor extrinsic-parameters, we evaluated the effect of dinaciclib on angiogenesis in the A2780 xenograft model, as transcriptional repressors such as triptolide have been reported to block angiogenesis [39]. Angiogenesis in A2780 tumors was measured by CD34 quantification of microvessel density. Following three dinaciclib treatments on the q4d schedule, a substantial reduction of microvessel density was observed that was similar to the antiangiogenesis KDR inhibitor, which was used as a positive control (compound B [19]) (Figure 5D). Dinaciclib's antiangiogenic effect was independent of tumor size (data not shown). These data suggest that while apoptosis induction is greatest in *MCL1:BCL-xL* high ratio tumors, inhibition of angiogenesis may be a contributing factor to the efficacy response of dinaciclib observed in all xenograft models. The association between tumor vascular density and dinaciclib anti-tumor effect will require further investigation.

## Discussion

We sought to investigate *in vitro* and *in vivo* biomarkers associated with dinaciclib response using exposure times which more closely mimic the clinical duration of exposure. Results from >250 solid tumor cell lines demonstrated differential cell line sensitivity that correlated with expression levels of the antiapoptotic BCL2-family member *BCL-xL*, the *MCL1:BCl-xL* mRNA ratio and *MCL1* gene copy number. These results are in agreement with reports using pan-CDK inhibitors such as flavopiridol and inhibitors of transcription such as triptolide [23]. Dinaciclib specifically induced apoptosis in cell lines expressing a high level of MCL1 relative to compensatory BCL-xL. The basis of this pro-apoptotic response is associated with the downregulation of MCL1 mRNA and protein that occurs after dinaciclib inhibition of CDK phosphorylation sites within the RNAP II CTD required for transcriptional function [9]. MCL1 is particularly sensitive to transcriptional arrest because of its short mRNA and protein half-life (30–100 min) [40], [41]. *In vitro* wash-out experiments showed that RNAP II Ser-2 phosphorylation and MCL1 protein levels rapidly recovered after dinaciclib removal and greater than 2 hours exposure is required for a robust apoptotic response in MCL1-dependent cell lines. As application of mRNA signatures to the clinical setting may be challenging, we sought to evaluate whether *MCL1* copy number correlated with sensitivity as this would provide a more binary readout for patient enrollment. Many of the *MCL1* amplified cell lines were identified to be MCL1-dependent by siRNA knockdown and sensitive to dinaciclib, suggesting that *MCL1* copy number may be a useful means for patient enrollment. One challenge using *MCL1* copy number as a patient enrichment strategy may be the limited dynamic range in copy number gain observed in human clinical samples (Figure S7). As such, a copy number cut-off of ≥4 would only make ∼1% of breast cancer patients identifiable by this strategy. However, reducing the threshold to *MCL1* copy number of ≥3 would allow for ∼8% of breast cancers to be eligible. The siRNA and dinaciclib results described here suggest that cell lines with copy number of ≥3 are sensitive and therefore may be a reasonable biomarker enrollment threshold, if this threshold can be accurately defined using available technology.

Not all *MCL1*-amplified cell lines were sensitive to *MCL1* siRNA treatment, but were sensitive to dinaciclib, suggesting that dinaciclib provides additional means to cell-killing beyond downregulation of MCL1 alone. Examples of such multivariate effects have been reported with dinaciclib's ability to also upregulate BIM [42]. We were however unable to observe similar BIM effects using shorter-term dinaciclib treatments *in vitro* and *in vivo* in responsive xenograft tumors (data not shown). These pleotropic effects resulting from a block in transcription are in part the result of dinaciclib targeting CDK1, 2 and 9 [8]. Dinaciclib's ability to induce apoptosis was supported by use of apoptotic-deficient cell lines. Additionally, these studies demonstrated that while dinaciclib can induce cell cycle arrest at G1/S and G2/M in 24 hr of treatment, this had minimal impact (<30%) on cell viability and was non-apparent with shorter treatments, even though these treatments were sufficient to decrease RNAP II phosphorylation and diminish MCL1 protein, which were rapidly reversible following dinaciclib wash-out.

Dinaciclib induction of tumor cell apoptosis was also observed *in vivo* in *MCL1:BCL-xL* high ratio tumors, but not in low ratio tumors. Validation that MCL1 downregulation is a major mechanism of dinaciclib's effect was further supported by observing the greatest efficacy effect in the highly *MCL1*-dependent NCI-H23 xenograft tumor model. This was observed in nonclinical studies using a dose that covered concentrations but slightly less duration than that observed in the clinic. While dinaciclib treatment caused tumor regression in the NCI-H23 xenograft model, the other three *MCL1:BCL-xL* high mRNA ratio xenograft models (COLO 320DM, A2780 and 22Rv1) showed only ∼40–50% tumor growth inhibition, despite apoptosis induction within the tumors.

While collectively these data support that apoptosis is an important mechanism of dinaciclib's effect, the antitumor response observed in the *MCL1:BCL-xL* low ratio tumors, which lacked evidence of apoptosis induction, suggested involvement of additional antitumor mechanisms. Since inhibitors of transcription have been reported to inhibit angiogenesis [39], we evaluated dinaciclib's effect on angiogenesis in a representative xenograft tumor model. In agreement with this hypothesis, decreased angiogenesis was observed in the one tumor model evaluated. Additional studies utilizing a range of tumor models are needed to evaluate this antiangiogenesis response as well as additional tumor intrinsic and extrinsic pathways. Such studies may be realized using an RNA-Seq approach in mouse xenograft tumor models where both human and mouse genes and pathways can be measured.

## Conclusions

In summary, our findings suggest that MCL1 copy number or the MCL1:BCL-xL mRNA ratio may provide a rational enrichment strategy for identifying cancer patients whose tumors are most likely to respond to dinaciclib. Additionally, while both apoptosis induction and antiangiogenesis likely contribute to dinaciclib's anti-tumor effect, the in vivo apoptosis response a priori will likely be enhanced through combination with drugs that downregulate or directly inhibit other BCL2-family members and thus may provide clinical benefit.

## Supporting Information

Figure S1 **Target engagement, MCL1 and cell viability responses to dinaciclib relative to the** ***MCL:BCL-xL*** **mRNA ratio.** (A) Immunoblot analysis of lysates prepared from 13 human cancer cell lines after 8 hr dinaciclib (100 nM) or DMSO treatment. Equal total protein amounts were loaded in order of high to low *MCL1:BCL-xL* mRNA ratio (see Table S1). (B) Effect of 18 hr dinaciclib (100 nM) treatment on cell viability (y axis) of 23 human cancer cell lines correlates with the *MCL1:BCL-xL* mRNA ratio (x axis). Plotted is the mean % viability remaining from 2–6 plates with cells in triplicate/plate. The seven cell lines used for xenograft tumor studies are highlighted (open triangles). *MCL1* and *BCL-xL* expression values were obtained from the Cancer Cell Line Encyclopedia.
(PDF)

Figure S2 **Effect of dinaciclib on antiapoptotic BCL2 members and apoptosis induction in A2780 and NCI-H23 cells.** (A) *BCL2* and *BCL-xL* mRNA levels in A2780 cells during the 5 hr dinaciclib (100 nM) treatment shown in Figure 2C. mRNA data were normalized to the geometric mean of *α-tubulin* and *GAPDH* mRNA levels. (B) Dinaciclib (100 nM) downregulates *MCL1* mRNA expression in NCI-H23 cells during a 5 hr treatment. Expression level is relative to α-tubulin. (C) Immunoblot analysis of NCI-H23 cells during the 5 hr time-course in (B), showing MCL1 protein downregulation after 2 hr treatment and induction of apoptosis as measured by cleaved PARP. α-tubulin is included as a loading control. (D) Immunoblot analysis of NCI-H23 cells treated with dinaciclib (100 nM) for 0, 2 or 8 hr (lanes 1, 2 and 6). After 2 hr treatment, dinaciclib was washed-out and cells were analyzed at subsequent 2 hr intervals with the cumulative times from t = 0 as indicated (lanes 3, 4 and 5).
(TIF)

Figure S3 **Dinaciclib and navitoclax exhibit synergistic cell killing in cell lines resistant to the single agents.** (A–D) Bliss synergy analysis graph of expected cell viability response to each single agent at specified concentrations (grid intersections) compared to observed (black balls) cell viability fractional response after 18 hr treatment in 8×8 dose escalation combination matrix of dinaciclib and navitoclax. The dotted lines correspond to the fractional viability of dinaciclib (left, rear) and navitoclax (right, rear) alone at the respective treatment doses. The Bliss synergy values are summarized in Table 1.
(TIF)

Figure S4 **Representative dinaciclib pharmacokinetics in mice and time-course of apoptosis in NCI-H23 and COLO-320DM xenograft tumors.** (A) Dinaciclib concentrations measured in whole blood collected at 0, 0.25, 0.5, 1, 2 and 4 hr after a single 40 mg/kg, i.p. administration to NCI-H2122 tumor-bearing CD1 female nude mice. Time 0 is a 1 hr vehicle treatment. Shown is the mean and standard deviation from 3 mice per timepoint. (B & C) Kinetics of apoptosis induction measured by cleaved-PARP fragment levels in protein lysates of NCI-H23 (B) and COLO 320DM (C) xenograft tumors resected at the indicated times following a single dinaciclib 40 mg/kg, i.p. injection. Cleaved-PARP counts represent the mean and standard deviation of 4–5 tumors per group. The corresponding immunoblot analysis of these studies is shown in Figure 5A.
(TIF)

Figure S5 **Apoptosis-induction by dinaciclib correlates with the** ***MCL1:BCL-xL*** **mRNA ratio in seven human xenograft tumor models.** Mice were implanted with cell suspensions or tumor fragments and given a single 40 mg/kg, i.p. injection of dinaciclib or vehicle when the mean tumor volumes reached 300–400 mg. All xenograft studies consisted of 5 mice/group with one tumor/mouse, except for 22Rv1 which had 4 tumors in the vehicle-only group. Tumors were resected at 6 hr post-dosing and lysates were analyzed by immunoblotting. The first and last lanes of each blot are lysates prepared from the corresponding cell line grown under tissue-culture (TC) conditions and treated with DMSO or dinaciclib (100 nM) for 8 hr, respectively. Equal total protein was loaded for each sample and α-tubulin was included as a loading control. Blot intensities are not normalized between xenograft models.
(TIF)

Figure S6 **Efficacy of dinaciclib in seven xenograft tumor models.** In each study, tumors were sized matched before dosing was initiated on day 1. (A–G) Points plotted are the mean tumor volumes and standard error of the mean (SEM) determined from bi-weekly caliper measurements (n = 10 mice per group, except n = 8 mice in the JIMT-1 dinaciclib group). Mice bearing NCI-H23 xenograft tumors were dosed twice weekly while all other xenograft models were dosed q4d with dinaciclib at 40 mg/kg, i.p. (filled squares) or vehicle (open squares). All statistics and analyses of efficacy were conducted by comparing to the vehicle control. Dinaciclib exhibited a statistically significant inhibition of tumor growth at the end of study for all xenograft models (p<0.05).
(TIF)

Figure S7 ***MCL1*** **gene copy number in human tumors.** (A) Copy number (CN) counts of *MCL1* measured across a variety of solid tumors. Human tumor data was obtained from Life Technologies - Oncomine Power Tools. (B) Frequency of *MCL1* amplification across various human tumors using CN of ≥4 and (C) CN threshold ≥3.
(TIF)

Table S1 High *MCL1:BCL-xL* mRNA ratio is associated with dinaciclib-induced apoptosis after 100 nM, 8 hr treatment.
(DOCX)

Table S2 Correlation of dinaciclib response to *MCL1:BCL-xL* mRNA ratio solid tumor cell lines by cancer type.
(DOCX)

Table S3 MCL1-dependence determined by siRNA knockdown.
(DOCX)

Table S4 Dinaciclib causes both G1/S and G2/M cell cycle arrest in apoptosis-deficient cells.
(DOCX)

Table S5 This file contains the percent (%) viability remaining values for 254 cell lines after 24 hr treatment with 100 nM dinaciclib. This file also lists the *MCL1* and *BCL-xL* mRNA expression levels (log10), the *MCL1:BCL-xL* mRNA ratio (log10), cancer type and source (vendor and catalog number) for each cell line.
(XLSX)
