# Supplementary figures and images for: MCL1 and BCL-xL Levels in Solid Tumors Are Predictive of Dinaciclib-Induced Apoptosis
Source: PLoS One. 2014 Oct 7;9(10):e108371. doi: 10.1371/journal.pone.0108371 (PMC4188521; doi:10.1371/journal.pone.0108371)

Figure S1

A

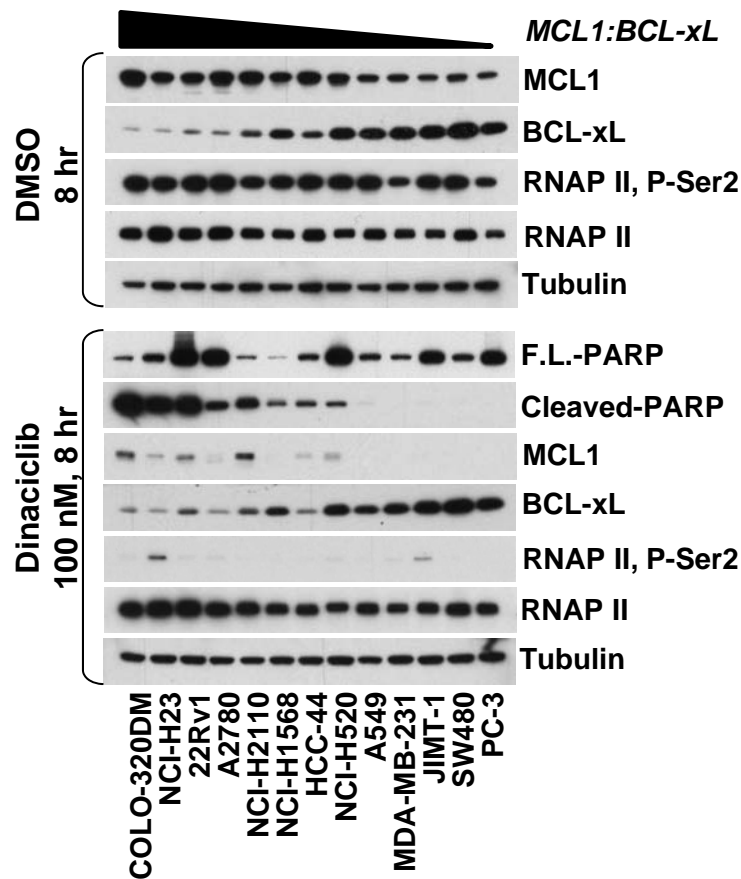

B

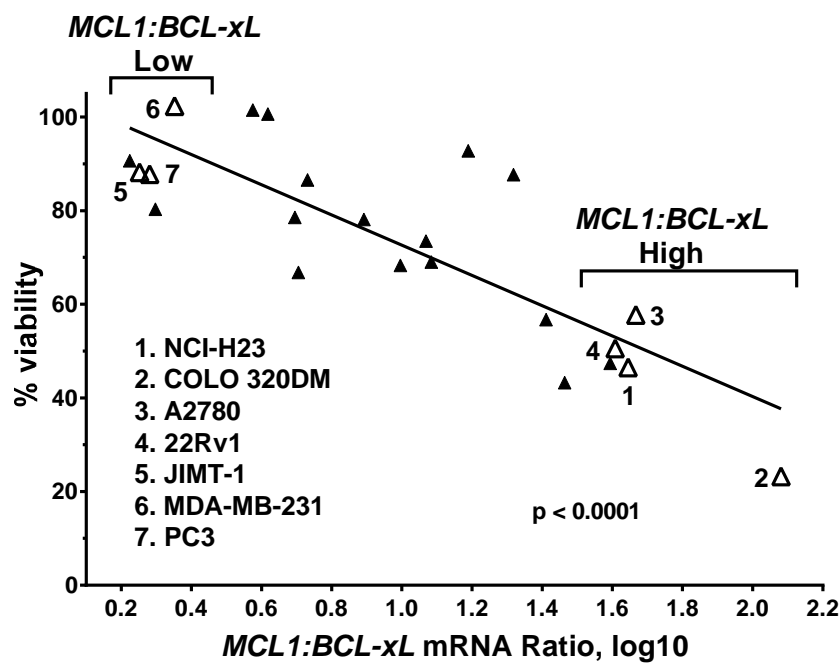

Supplement: Figure S1 — Target engagement, MCL1 and cell viability responses to dinaciclib relative to the MCL:BCL-xL mRNA ratio. (A) Immunoblot analysis of lysates prepared from 13 human cancer cell lines after 8 hr dinaciclib (100 nM) or DMSO treatment. Equal total protein amounts were loaded in order of high to low MCL1:BCL-xL mRNA ratio (see Table S1). (B) Effect of 18 hr dinaciclib (100 nM) treatment on cell viability (y axis) of 23 human cancer cell lines correlates with the MCL1:BCL-xL mRNA ratio (x axis). Plotted is the mean % viability remaining from 2–6 plates with cells in triplicate/plate. The seven cell lines used for xenograft tumor studies are highlighted (open triangles). MCL1 and BCL-xL expression values were obtained from the Cancer Cell Line Encyclopedia. (PDF) [file pone.0108371.s001.pdf]

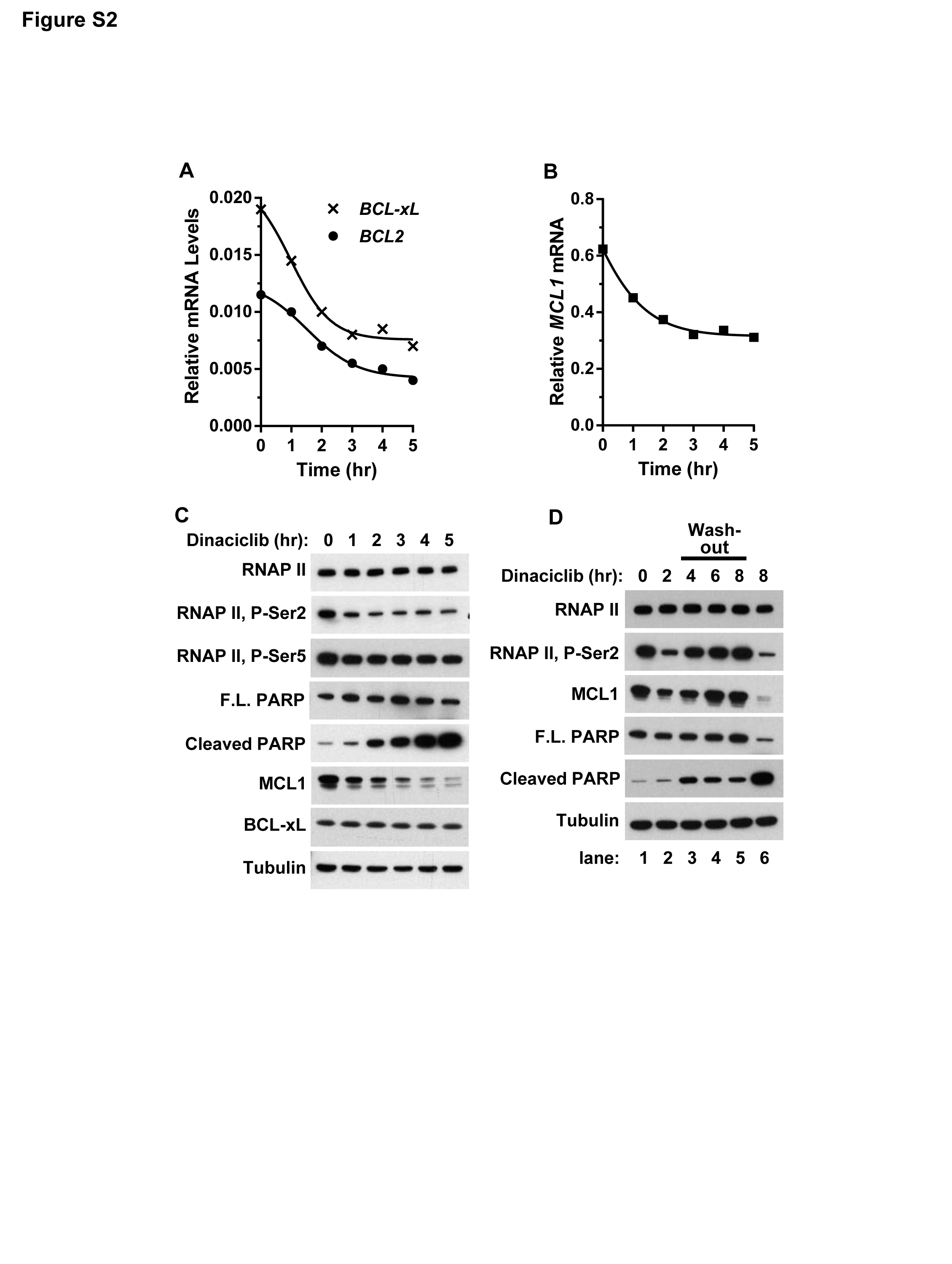

Supplement: Figure S2 — Effect of dinaciclib on antiapoptotic BCL2 members and apoptosis induction in A2780 and NCI-H23 cells. (A) BCL2 and BCL-xL mRNA levels in A2780 cells during the 5 hr dinaciclib (100 nM) treatment shown in Figure 2C. mRNA data were normalized to the geometric mean of α-tubulin and GAPDH mRNA levels. (B) Dinaciclib (100 nM) downregulates MCL1 mRNA expression in NCI-H23 cells during a 5 hr treatment. Expression level is relative to α-tubulin. (C) Immunoblot analysis of NCI-H23 cells during the 5 hr time-course in (B), showing MCL1 protein downregulation after 2 hr treatment and induction of apoptosis as measured by cleaved PARP. α-tubulin is included as a loading control. (D) Immunoblot analysis of NCI-H23 cells treated with dinaciclib (100 nM) for 0, 2 or 8 hr (lanes 1, 2 and 6). After 2 hr treatment, dinaciclib was washed-out and cells were analyzed at subsequent 2 hr intervals with the cumulative times from t = 0 as indicated (lanes 3, 4 and 5). (TIF) [file pone.0108371.s002.tif]

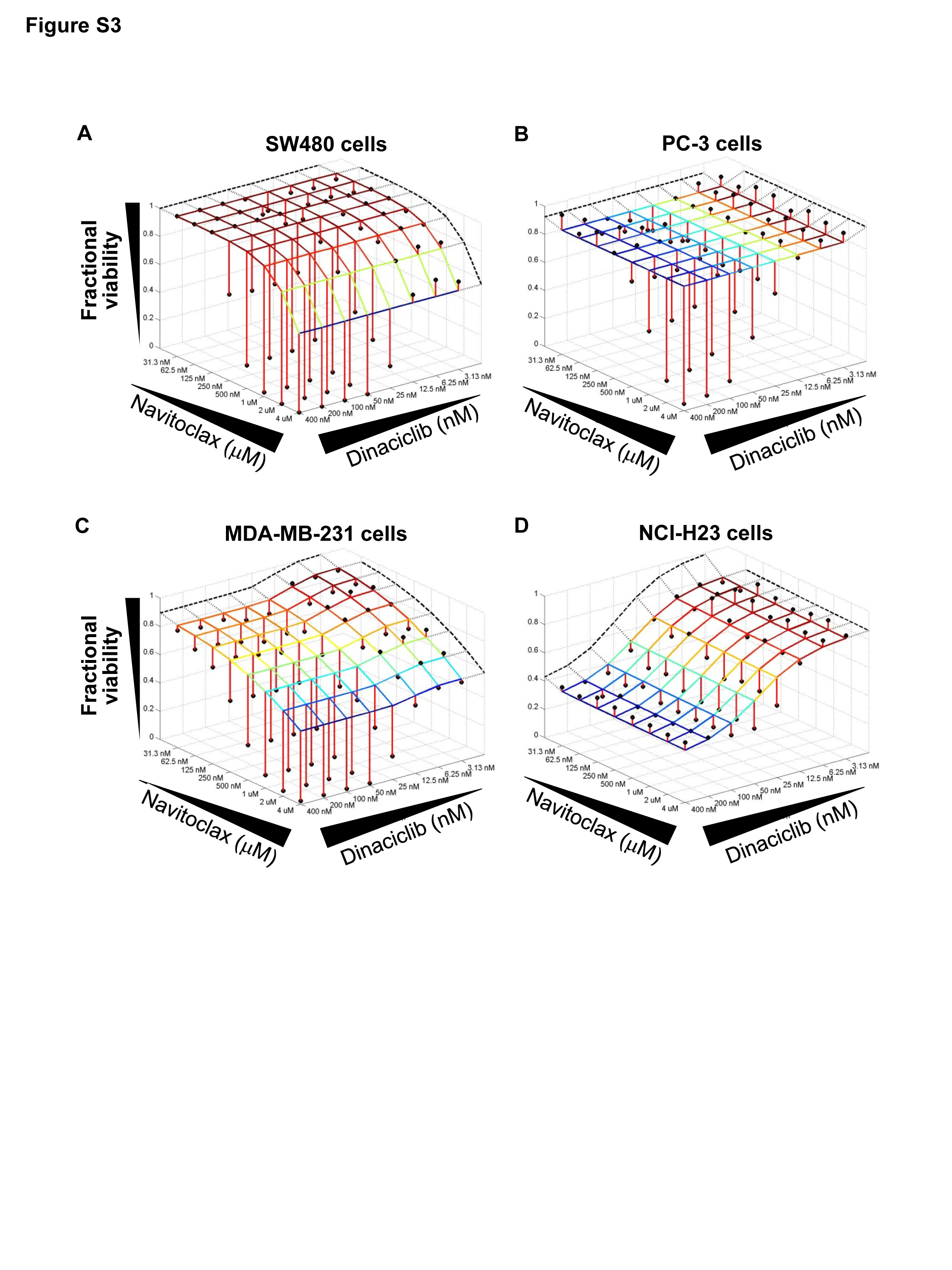

Supplement: Figure S3 — Dinaciclib and navitoclax exhibit synergistic cell killing in cell lines resistant to the single agents. (A–D) Bliss synergy analysis graph of expected cell viability response to each single agent at specified concentrations (grid intersections) compared to observed (black balls) cell viability fractional response after 18 hr treatment in 8×8 dose escalation combination matrix of dinaciclib and navitoclax. The dotted lines correspond to the fractional viability of dinaciclib (left, rear) and navitoclax (right, rear) alone at the respective treatment doses. The Bliss synergy values are summarized in Table 1. (TIF) [file pone.0108371.s003.tif]

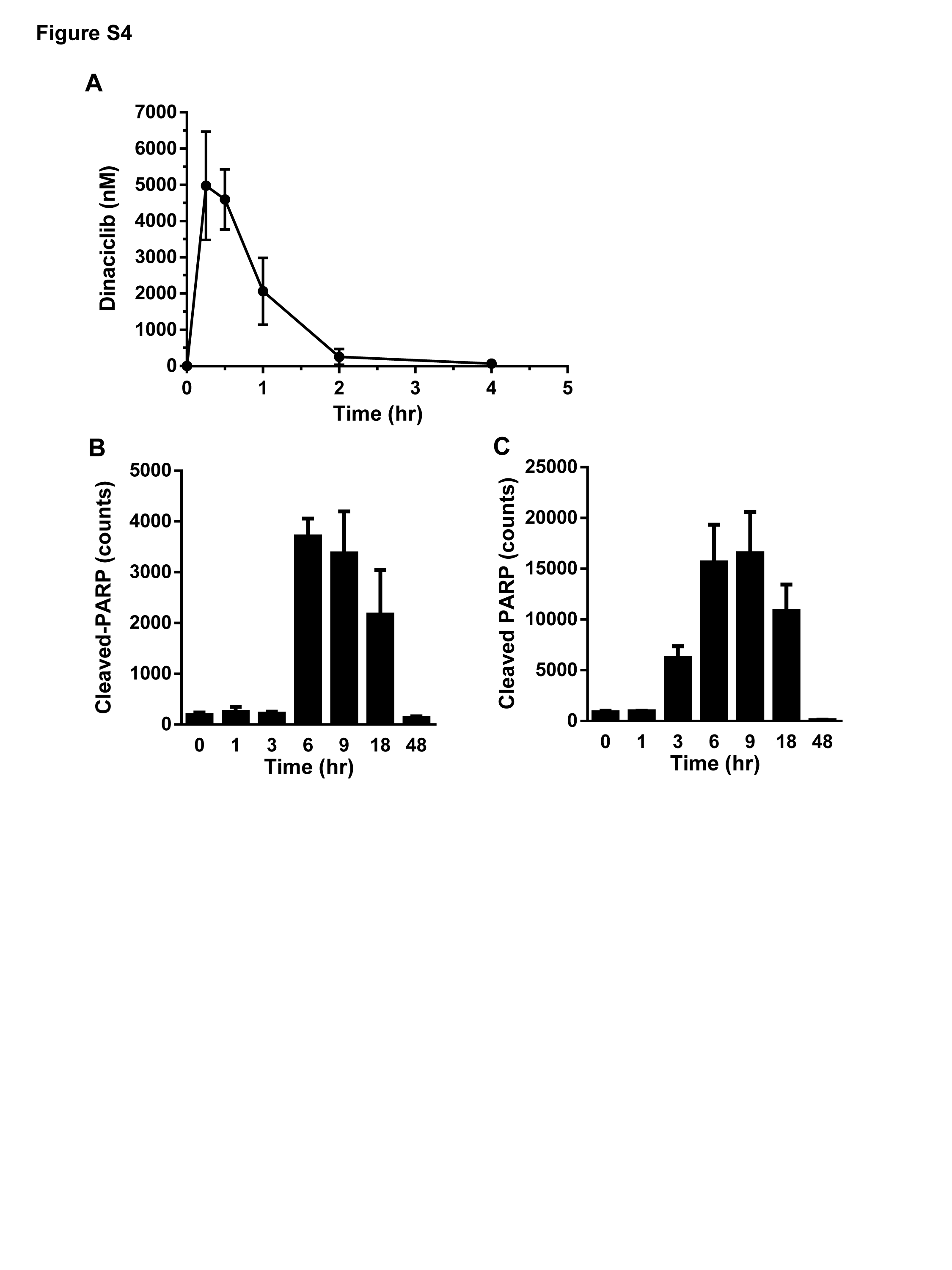

Supplement: Figure S4 — Representative dinaciclib pharmacokinetics in mice and time-course of apoptosis in NCI-H23 and COLO-320DM xenograft tumors. (A) Dinaciclib concentrations measured in whole blood collected at 0, 0.25, 0.5, 1, 2 and 4 hr after a single 40 mg/kg, i.p. administration to NCI-H2122 tumor-bearing CD1 female nude mice. Time 0 is a 1 hr vehicle treatment. Shown is the mean and standard deviation from 3 mice per timepoint. (B & C) Kinetics of apoptosis induction measured by cleaved-PARP fragment levels in protein lysates of NCI-H23 (B) and COLO 320DM (C) xenograft tumors resected at the indicated times following a single dinaciclib 40 mg/kg, i.p. injection. Cleaved-PARP counts represent the mean and standard deviation of 4–5 tumors per group. The corresponding immunoblot analysis of these studies is shown in Figure 5A. (TIF) [file pone.0108371.s004.tif]

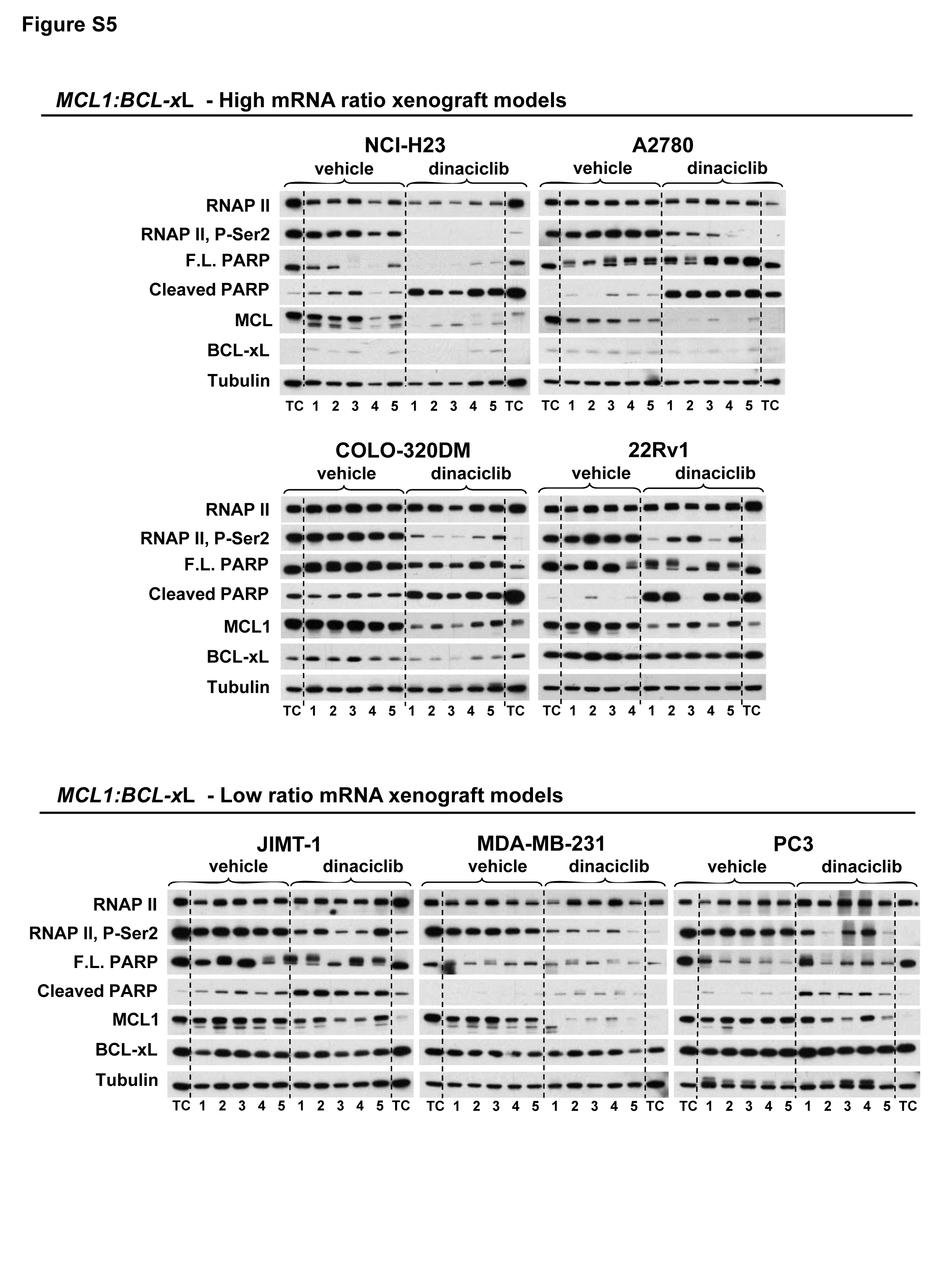

Supplement: Figure S5 — Apoptosis-induction by dinaciclib correlates with the MCL1:BCL-xL mRNA ratio in seven human xenograft tumor models. Mice were implanted with cell suspensions or tumor fragments and given a single 40 mg/kg, i.p. injection of dinaciclib or vehicle when the mean tumor volumes reached 300–400 mg. All xenograft studies consisted of 5 mice/group with one tumor/mouse, except for 22Rv1 which had 4 tumors in the vehicle-only group. Tumors were resected at 6 hr post-dosing and lysates were analyzed by immunoblotting. The first and last lanes of each blot are lysates prepared from the corresponding cell line grown under tissue-culture (TC) conditions and treated with DMSO or dinaciclib (100 nM) for 8 hr, respectively. Equal total protein was loaded for each sample and α-tubulin was included as a loading control. Blot intensities are not normalized between xenograft models. (TIF) [file pone.0108371.s005.tif]

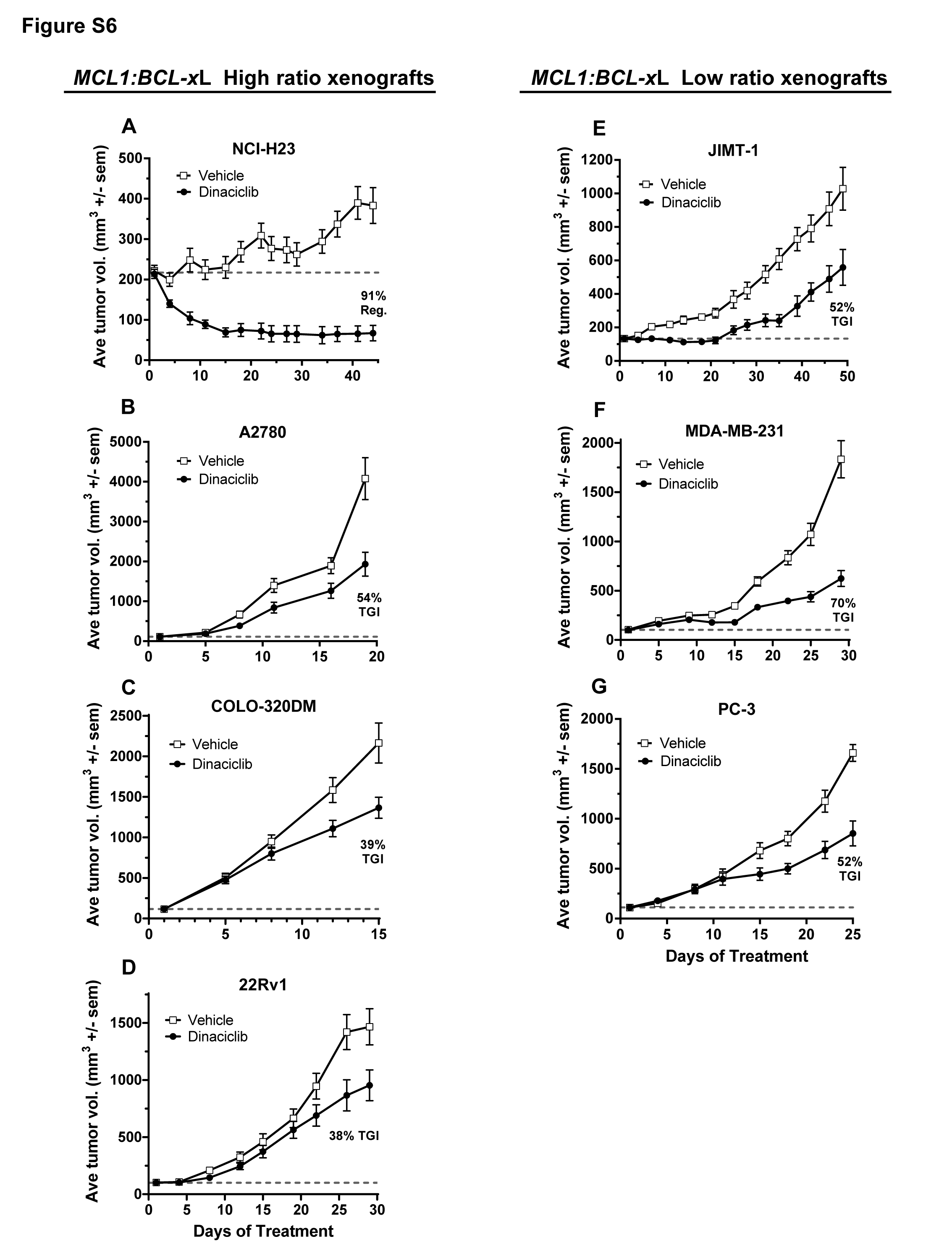

Supplement: Figure S6 — Efficacy of dinaciclib in seven xenograft tumor models. In each study, tumors were sized matched before dosing was initiated on day 1. (A–G) Points plotted are the mean tumor volumes and standard error of the mean (SEM) determined from bi-weekly caliper measurements (n = 10 mice per group, except n = 8 mice in the JIMT-1 dinaciclib group). Mice bearing NCI-H23 xenograft tumors were dosed twice weekly while all other xenograft models were dosed q4d with dinaciclib at 40 mg/kg, i.p. (filled squares) or vehicle (open squares). All statistics and analyses of efficacy were conducted by comparing to the vehicle control. Dinaciclib exhibited a statistically significant inhibition of tumor growth at the end of study for all xenograft models (p<0.05). (TIF) [file pone.0108371.s006.tif]

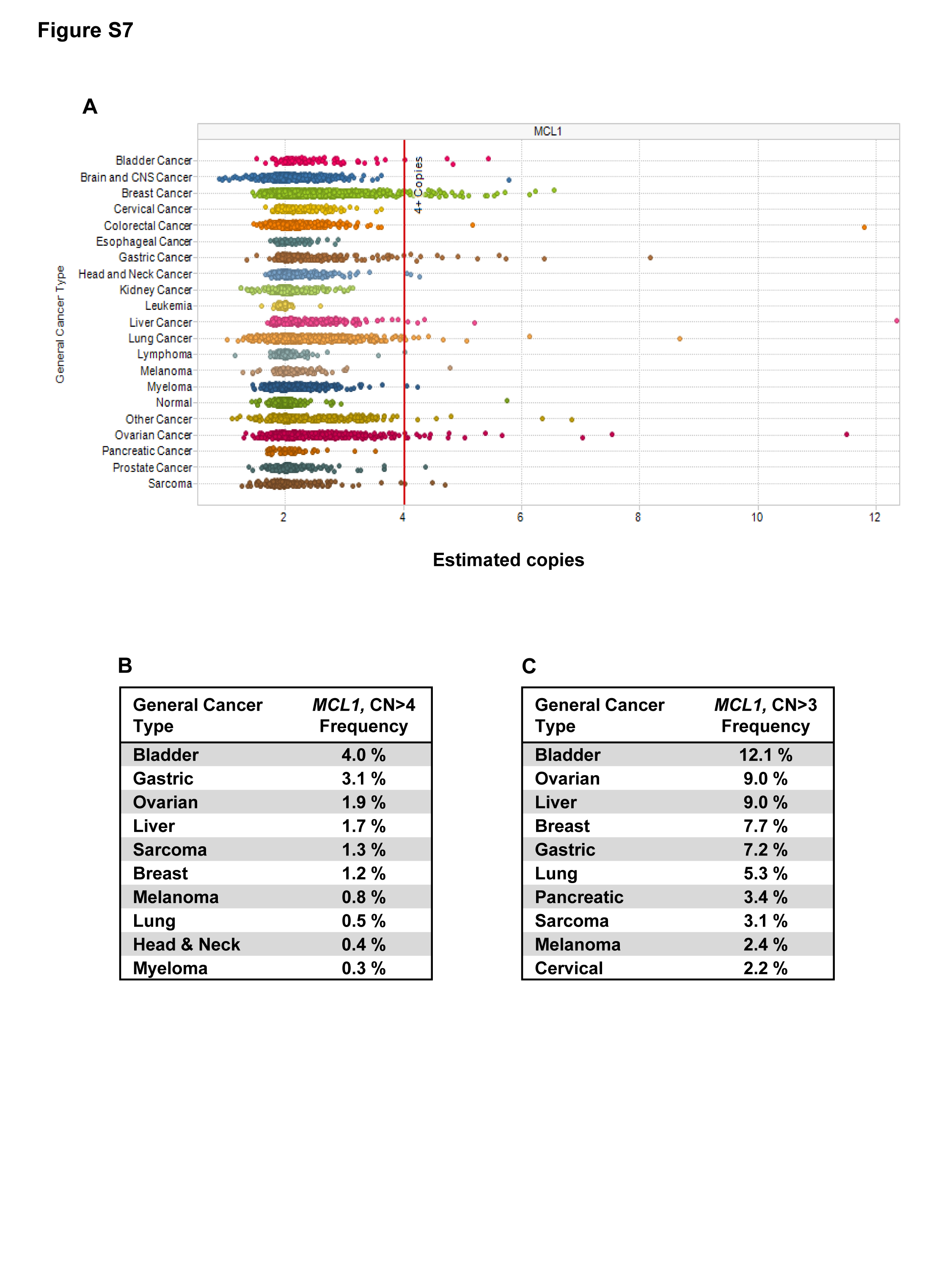

Supplement: Figure S7 — MCL1 gene copy number in human tumors. (A) Copy number (CN) counts of MCL1 measured across a variety of solid tumors. Human tumor data was obtained from Life Technologies - Oncomine Power Tools. (B) Frequency of MCL1 amplification across various human tumors using CN of ≥4 and (C) CN threshold ≥3. (TIF) [file pone.0108371.s007.tif]
