# Supplementary material for: MCL1 and BCL-xL Levels in Solid Tumors Are Predictive of Dinaciclib-Induced Apoptosis
Source: PLoS One. 2014 Oct 7;9(10):e108371. doi: 10.1371/journal.pone.0108371 (PMC4188521; doi:10.1371/journal.pone.0108371)
Supplement: Table S1 — High MCL1:BCL-xL mRNA ratio is associated with dinaciclib-induced apoptosis after 100 nM, 8 hr treatment. (DOCX) [file pone.0108371.s008.docx]

**Table S1**. High *MCL1:BCL-xL* mRNA ratio is associated with dinaciclib-induced apoptosis after 100 nM, 8 hr treatment

| **Cell line** | **Cancer origin** | ***MCL1* mRNA^1^ (log10)** | ***BCL-xL* mRNA^1^ (log10)** | ***MCL1:BCL-xL* mRNA ratio^1^ (log10)** | **Cleaved PARP product^2^** |
| --- | --- | --- | --- | --- | --- |
| COLO 320DM | Colon | 3.59 | 1.51 | 2.08 | 35068 |
| A2780 | Ovarian | 3.42 | 1.76 | 1.67 | 3014 |
| NCI-H23 | NSCLC | 3.40 | 1.75 | 1.64 | 16515 |
| NCI-H2110 | NSCLC | 3.29 | 1.67 | 1.63 | 2727 |
| HCC1954 | Breast | 3.42 | 1.82 | 1.59 | 2609 |
| NCI-H1568 | NSCLC | 3.50 | 2.08 | 1.41 | 3254 |
| NCI-H1437 | NSCLC | 3.26 | 1.80 | 1.46 | 3159 |
| 22Rv1 | Prostate | 3.17 | 1.57 | 1.61 | 21992 |
| HCC-44 | NSCLC | 3.37 | 2.00 | 1.37 | 2007 |
| NCI-H460 | LCLC | 3.20 | 1.82 | 1.38 | 758 |
| NCI-H1792 | Lung | 3.21 | 2.12 | 1.08 | 1823 |
| NCI-H522 | NSCLC | 3.19 | 2.00 | 1.19 | 1232 |
| NCI-H1975 | NSCLC | 3.38 | 2.31 | 1.07 | 894 |
| NCI-H2122 | NSCLC | 3.43 | 2.44 | 1.00 | 970 |
| NCI-H322 | NSCLC | 3.19 | 2.23 | 0.96 | 1 |
| HCC827 | Lung | 3.17 | 2.28 | 0.89 | 26 |
| NCI-H1838 | NSCLC | 3.48 | 2.75 | 0.73 | 1043 |
| NCI-H520 | Lung | 3.22 | 2.52 | 0.70 | 1475 |
| HCT-15 | Colon | 3.17 | 2.46 | 0.71 | 595 |
| A549 | Lung | 3.22 | 2.52 | 0.69 | 273 |
| NCI-H1793 | NSCLC | 3.17 | 2.59 | 0.57 | 1 |
| SW1573 | Lung | 3.00 | 2.39 | 0.62 | 163 |
| MDA-MB-231 | Breast | 3.14 | 2.79 | 0.35 | 7 |
| PC-3 | Prostate | 3.05 | 2.77 | 0.28 | 1 |
| SW620 | Colon | 3.02 | 2.72 | 0.30 | 23 |
| JIMT-1 | Breast | 2.75 | 2.50 | 0.25 | 129 |
| SW480 | Colon | 2.95 | 2.73 | 0.22 | 46 |

^1^ Information source: Cancer Cell Line Encyclopedia converted from log 2 to log10

^2^ Cleaved PARP product (relative density) quantified from immunoblot autoradiographs

NSCLC, non-small cell lung cancer; LCLC, large cell lung cancer
