# Supplementary material for: MCL1 and BCL-xL Levels in Solid Tumors Are Predictive of Dinaciclib-Induced Apoptosis
Source: PLoS One. 2014 Oct 7;9(10):e108371. doi: 10.1371/journal.pone.0108371 (PMC4188521; doi:10.1371/journal.pone.0108371)
Supplement: Table S2 — Correlation of dinaciclib response to MCL1:BCL-xL mRNA ratio solid tumor cell lines by cancer type. (DOCX) [file pone.0108371.s009.docx]

**Table S2**. Correlation of dinaciclib response to *MCL1:BCL-xL* mRNA

ratio in solid tumor cell lines by cancer type

| **Tumor Type** | **# Cell Lines** | ***p-value*** |
| --- | --- | --- |
| Ovary | 33 | 0.0003 |
| Brain | 16 | 0.0007 |
| Lung | 46 | 0.0037 |
| Head & Neck | 6 | 0.0068 |
| Kidney | 7 | 0.0152 |
| Endometrium | 19 | 0.0172 |
| Breast | 25 | 0.0214 |
| Sarcoma | 13 | 0.0471 |
| Gastric | 12 | 0.0660 |
| Colon | 26 | 0.0892 |
| Melanoma | 35 | 0.5674 |
| Pancreas | 10 | 0.7478 |
| Liver | 2 | nd |
| Prostate | 4 | nd |
| **All Tumors** | **254** | **<0.0001** |
| nd, not determined | | |
