# Supplementary material for: MCL1 and BCL-xL Levels in Solid Tumors Are Predictive of Dinaciclib-Induced Apoptosis
Source: PLoS One. 2014 Oct 7;9(10):e108371. doi: 10.1371/journal.pone.0108371 (PMC4188521; doi:10.1371/journal.pone.0108371)
Supplement: Table S3 — MCL1-dependence determined by siRNA knockdown. (DOCX) [file pone.0108371.s010.docx]

**Table S3**. MCL1-dependence determined by siRNA knockdown

| **Cell Line** | **Cancer origin** | **Dinaciclib sensitive^1^** | **MCL1 dependent^2^** | ***MCL1* copy number^3^** | ***MCL1: BCL-xL* mRNA ratio^4^** |
| --- | --- | --- | --- | --- | --- |
| CAL-120 | Breast | N | N | 2 | 0.21 |
| SW1271 | SCLC | N | N | 1 | 0.44 |
| T47D | Breast | N | N | 3 | 0.58 |
| HCC1806 | Breast | N | N | 2 | 0.62 |
| A549 | NSCLC | N | N | 2 | 0.69 |
| NCI-H69 | SCLC | N | N | 2 | 0.91 |
| NCI-H322 | NSCLC | N | N | 3 | 0.96 |
| NCI-H2122 | NSCLC | N | N | 3 | 1.00 |
| NCI-H1975 | NSCLC | N | N | 3 | 1.07 |
| NCI-H522 | NSCLC | N | N | 3 | 1.19 |
| NCI-H82 | SCLC | Y | Y | 2 | 1.30 |
| MCF-7 | Breast | Y | N | 4 | 1.22 |
| NCI-H1568 | NSCLC | Y | N | 7 | 1.41 |
| SHP77 | SCLC | Y | Y | 3 | 1.56 |
| NCI-H2110 | NSCLC | Y | N | 4 | 1.63 |
| NCI-H23 | NSCLC | Y | Y | 3 | 1.64 |
| NCI-H1395 | Lung | Y | N | 7 | 1.36 |
| HCC1187 | Breast | Y | Y | 5 | 1.76 |

^1^ Sensitive, >70% decrease in cell viability after 24 hr dinaciclib (100 nM) treatment

^2^ Dependent, >70% decrease in cell viability following 24 hr *MCL1* siRNA transfection

^3^ Information source: Cancer Cell Line Encyclopedia

^4^ Information source: Cancer Cell Line Encyclopedia converted from log 2 to log10

Y/N, yes/no; NSCLC, non-small cell lung cancer; SCLC, small cell lung cancer
