# Supplementary material for: MCL1 and BCL-xL Levels in Solid Tumors Are Predictive of Dinaciclib-Induced Apoptosis
Source: PLoS One. 2014 Oct 7;9(10):e108371. doi: 10.1371/journal.pone.0108371 (PMC4188521; doi:10.1371/journal.pone.0108371)
Supplement: Table S4 — Dinaciclib causes both G1/S and G2/M cell cycle arrest in apoptosis-deficient cells. (DOCX) [file pone.0108371.s011.docx]

**Table S4**. Dinaciclib causes both G1/S and G2/M cell cycle arrest in apoptosis-deficient cells

|  |  | **Cells in Cell Cycle (%)** | | | |
| --- | --- | --- | --- | --- | --- |
| **Cell Line** | **Treatment** | **Sub-G1** | **G1** | **S** | **G2/M** |
|  | Control | 2 | 45 | 34 | 19 |
| CA46 | Dinaciclib | 6 | 58 | 12 | 24 |
|  | Paclitaxel | 3 | 10 | 13 | 74 |
|  | Control | 11 | 48 | 26 | 15 |
| Daudi | Dinaciclib | 16 | 45 | 16 | 23 |
|  | Paclitaxel | 11 | 21 | 20 | 39 |
| DU145 | Control | 1 | 63 | 20 | 17 |
|  | Dinaciclib | 1 | 75 | 6 | 17 |
| KNS62 | Control | 0 | 54 | 22 | 20 |
|  | Dinaciclib | 1 | 69 | 9 | 21 |
|  | Control | 3 | 68 | 19 | 10 |
| Kasumi-1 | Dinaciclib | 65 | 14 | 15 | 3 |
|  | Paclitaxel | 7 | 39 | 23 | 30 |

Percent cells at cell cycle phases based on DNA content after 24 hr dinaciclib (100 nM) or paclitaxel (3 μM) treatments
